# Preparation and evaluation of ultrasound‐mediated dual‐targeted theragnostic systems utilising phase‐changeable polymeric nanodroplets on the integrin α_ν_β_3_ overexpressed breast cancer

**DOI:** 10.1002/ctm2.607

**Published:** 2021-10-14

**Authors:** Na Li, Shaobo Duan, Yiwei Wang, Linlin Zhang, Yongqing Chen, Juan Zhang, Ruiqing Liu, Yaqiong Li, Luwen Liu, Shanshan Ren, Ye Zhang, Yuqi Guo, Zhenyu Ji, Lianzhong Zhang

**Affiliations:** ^1^ Henan Provincial People's Hospital People's Hospital of Henan University, People's Hospital of Zhengzhou University Zhengzhou PR China; ^2^ Henan International Joint Laboratory for Gynecological Oncology and Nanomedicine Henan Provincial People's Hospital, People's Hospital of Zhengzhou University Zhengzhou PR China; ^3^ Institute of Medical and Pharmaceutical Sciences Zhengzhou University Zhengzhou PR China

To the Editor:

Breast cancer (BC) with overexpressed integrin α_ν_β_3_ often indicates a high rate of metastasis and recurrence.^1^ Therefore, targeted theragnostic systems are urgently required.^2^, ^3^ Cyclo(Arg‐Gly‐Asp‐d‐Tyr‐Lys) peptide (cRGD) ligand‐modified polymeric perfluorocarbon (PFC)‐based nanodroplet ultrasound contrast agent (UCA)^4^ can extravasate in, selectively adhere to, and accumulate in BC tissues due to their nanoscale size, ultrasound (US)‐mediated passive targeting and cRGD‐mediated active targeting properties. Under US irradiation in situ, they can undergo a phase shift, produce stable and inertial cavitation, induce useful US bioeffects, and provide enhanced sonography and improved therapeutic effects^5^, ^6^ that are ideal for targeted theragnostic systems. Here, we explored a dual‐targeted US‐responsive nanodroplet. Loaded with doxorubicin (DOX), the targeted nanodroplets led to enhanced US imaging and improved therapeutic effects, displaying excellent therapeutic efficacy.

As shown in Figure 1A, a novel dual‐targeted theragnostic UCA (DTTUCA), namely, cRGD‐modified poly(ethyleneglycol)‐poly(caprolactone) @ DOX (cRGD‐PEG‐PCL@DOX) nanodroplet, was constructed by self‐assembly and ultrasonic emulsification.^7^ The red fluorescence (FL) shells of DOX confirmed the successful construction of drug‐loaded DTTUCA and untargeted nanodroplets. All the nanodroplets were typically spherical and uniformly distributed within a relatively narrow range of <500 nm; transmission electron microscopy (TEM) and dynamic light scattering (DLS) results are given in Figure 1 and Table S1. Once the polymeric nano‐micelles were loaded with perfluorohexane (PFH), their diameters increased, indicating the successful preparation of polymeric shell‐based nanodroplets. According to the Laplace pressure Equation (1), small nanodroplets would need higher temperatures to undergo phase translation^8^; therefore, the nanodroplets remained relatively stable at 4°C (Figure S2). With low‐intensity focused ultrasound (LIFU) irradiation, DTTUCA expanded rapidly, indicating acoustic droplet vaporisation (ADV) and stable cavitation (Figure 1E and Figure S5).

(1) $$P_{L}=4\cdot \delta /d,$$

where *P*_L_ represents Laplace pressure, δ represents surface tension and d represents nanodroplet diameter.

**FIGURE 1:**
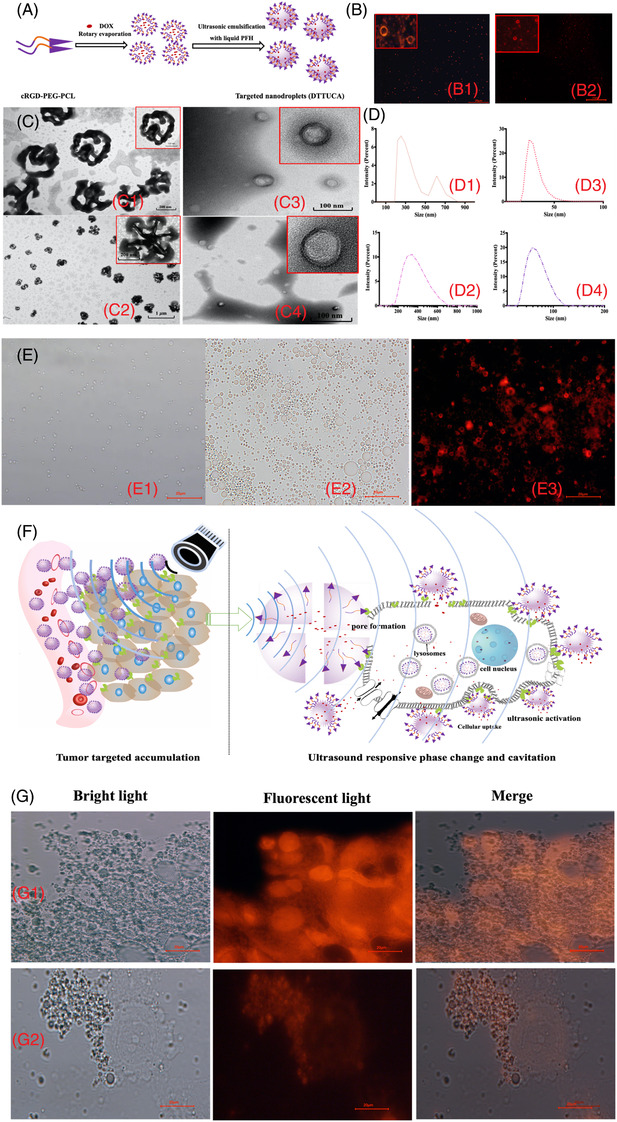
(A) Illustrations of preparation methods for dual‐targeted theragnostic ultrasound contrast agent (DTTUCA) nanodroplets and the untargeted nanoparticles. (B) Fluorescent images of DOX‐loaded DTTUCA and untargeted nanodroplets taken by fluorescent microscope. (C) Morphology studies (transmission electron microscopy results) for DTTUCA nanodroplets (a), mPEG_2k_‐PCL@DOX nanodroplets (b), cRGD‐PEG_2k_‐PCL micelles (c), and mPEG_2k_‐PCL micelles (d). (D) Size distribution studies (DLS measurements) for DTTUCA nanodroplets (a), mPEG_2k_‐PCL@DOX nanodroplets (b), cRGD‐PEG_2k_‐PCL micelles (c), and mPEG_2k_‐PCL micelles (d). (E) Study of ultrasound stimuli responsibility in vitro: (E1) bright light image of DTTUCA nanodroplets, (E2) bright light image of DTTUCA nanodroplets with LIFU2 irradiation for 15 s, (E3) fluorescent image of DTTUCA nanodroplets with LIFU2 irradiation for 15 s, all these pictures were taken by fluorescent microscope. (F) Schematic diagram depicting DTTUCA‐induced ultrasonic cavitation, promoted penetration, targeted drug release and enhanced cellular uptake resulting in efficient and precise therapeutic. (G) Study of the selectively adhering: representative microphotographs of DTTUCA and untargeted mPEG_2k_‐PCL@DOX nanodroplets co‐incubated with MCF‐7 cells for 40 min, group (G1) DTTUCA nanodroplets, and group (G2) untargeted mPEG_2k_‐PCL@DOX nanodroplets

The biomedical effects and acoustically responsive phase shift of DTTUCA is schematically depicted in Figure 1F. Indeed, DTTUCA selectively adhered to integrin α_V_β_3_ overexpressing BC cells (Figure 1G, Figures S4 and S6), as confirmed by the FL intensity analysis results shown in Tables S2 and S3. cRGD‐mediated cellular uptake enhancement and LIFU‐triggered acoustic cellular bioeffects confirmed that DTTUCA could substantially promote cellular uptake and inhibit tumour proliferation (Figure 2). Compared to the monomethoxy poly(ethylene glycol) (mPEG)‐PCL group, the DTTUCA group with cRGD ligand showed higher cellular uptake and lower cell viability. The relative FL intensity of DTTUCA with LIFU4 irradiation, untargeted nanodroplets and DOX·HCl was 22 282 ± 32 au, 1293 ± 40 au and 109 ± 23 au against MCF‐7 cells, respectively. As shown in Figure 2 and Figures S7, S8 and S20, LIFU stimulus evidently improved the cellular uptake and cytotoxicity of DTTUCA and the untargeted nanodroplets; Increasing LIFU power from one to four accelerated DOX translocation from the nuclear membrane into the nucleus^9^; however, without PFH nanodroplets, there was negligible effect. Although the biocompatible materials showed little cytotoxicity (Figure S3), cell viability decreased and the acoustic intensity of LIFU increased. DTTUCA nanodroplets with LIFU4 showed the highest cytotoxicity across all groups, which could be due to the US‐responsive phase shift and stable and inertial cavitation of the nanodroplets. Thus, the cRGD ligand, together with LIFU irradiation, could substantially promote selective adherence, cellular uptake and cytotoxicity of DTTUCA in vitro.

**FIGURE 2:**
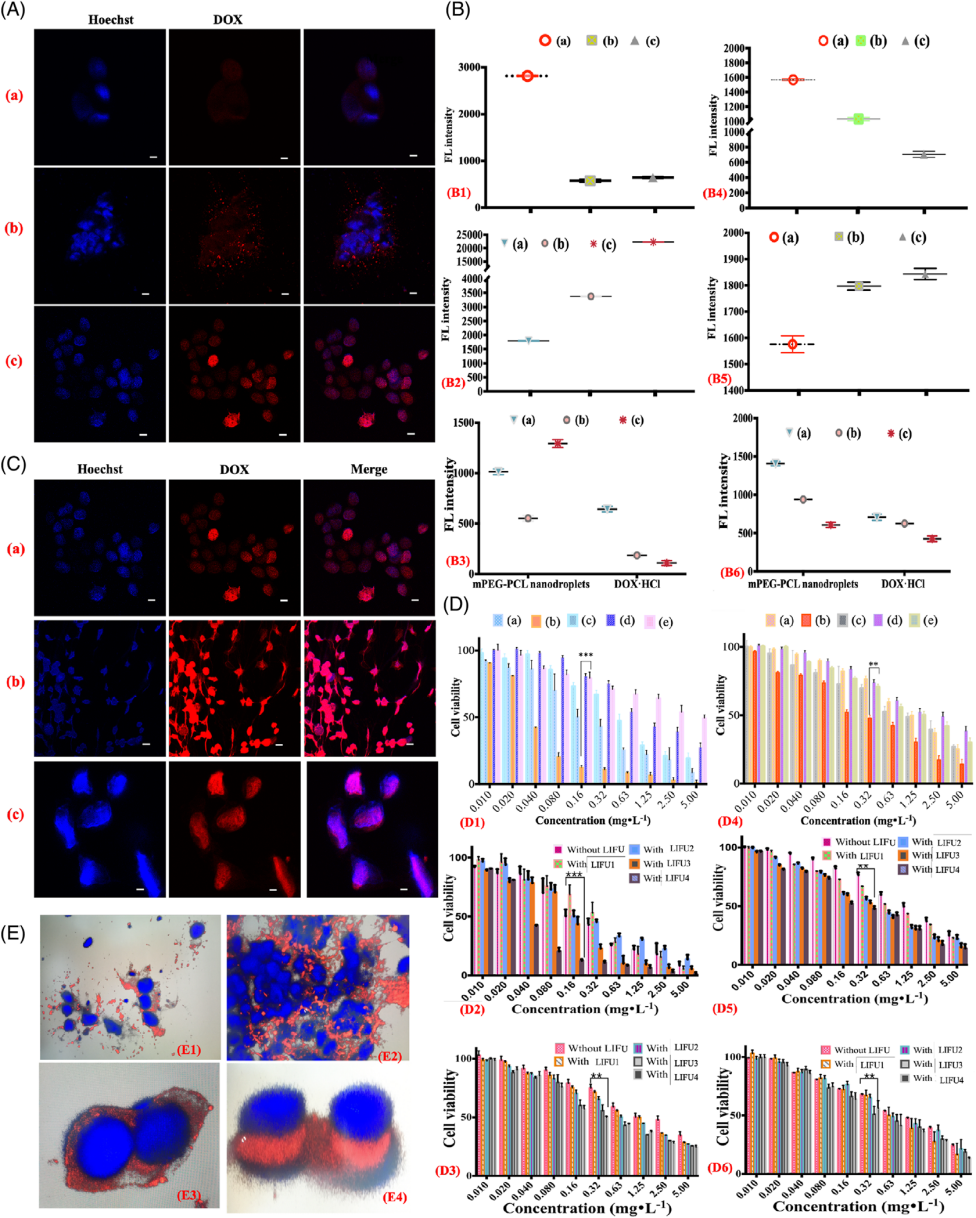
(A) Study of targetability: confocal laser scanning microscopy imaging (CLSM) of MCF‐7 cells that were co‐incubated with DOX•HCl (a), mPEG_2k_‐PCL@DOX micelles (b) and cRGD‐PEG_2k_‐PCL@DOX micelles (c) for 40 min. (B) Cellular uptake researches via flow cytometry (FCM) against MCF‐7 cells (B1, B2 and B3) and 4T1 cells (B4, B5 and B6); B1 and B4 were fluorescence (FL) intensity of cRGD‐PEG_2k_‐PCL nanomicelles (a), mPEG_2k_‐PCL nanomicelles (b) and DOX•HCl (c); B2 and B5 were FL intensity of dual‐targeted theragnostic ultrasound contrast agent (DTTUCA) nanodroplets (a), DTTUCA nanodroplets with LIFU3 irradiation (b) and DTTUCA nanodroplets with LIFU4 irradiation (c) against MCF‐7 cells and 4T1 cells; B3 and B6 were FL intensity of mPEG_2k_‐PCL nanodroplets and DOX•HCl against MCF‐7 cells (B3) and 4T1 cells (B6) for without LIFU (a), with LIFU3 irradiation (b) and LIFU4 irradiation (c). (C) Ultrasound stimuli responsive researches: CLSM of cRGD‐PEG_2k_‐PCL@DOX micelles (a), DTTUCA nanodroplets with LIFU1 (b) and DTTUCA nanodroplets with LIFU3 (c). (D) CCK‐8 results of MCF‐7 cells (D1, D2 and D3) and 4T1 cells (D4, D5 and D6): D1 and D4 were cell viabilities of DTTUCA (a), DTTUCA with LIFU4 irradiation (b), cRGD‐PEG_2k_‐PCL@DOX micelles (c), mPEG_2k_‐PCL@DOX micelles (d) and DOX•HCl (e) with 24 h co‐incubations; D2,D5 and D3,D6 were cell viabilities for 24 h co‐incubations of DTTUCA and untargeted nanodroplets together with different levels of LIFU irradiation, respectively. *p < .05; **p < .01; ***p < .001. (E) 3D pictures of the GLSM for DTTUCA nanodroplets incubation with MCF‐7 cells for 40 min under different levels of LIFU exposures. E1–E4, respectively, correspond to LIFU1–LIFU4 irradiations. The scale bars were 20 μm in all the images. The blue FL was Hoechst that showed the cell nucleus ranges, the red FL was DOX FL probe that showed the location of DTTUCA or DOX itself, and all the powers of the LIFU1 to LIFU4 were 0.5, 1.5, 2.5 and 3.5 W/cm^2^

UCA‐based enhanced US imaging of DTTUCA was qualitatively and quantitatively analysed in vitro (Figure 3). As shown in Figure 3A–C, DTTUCA could produce contrast‐enhanced ultrasound (CEUS) images at high mechanical index (MI) values under low fundamental frequency and at low MI values under high fundamental frequency. In contrast, water could not produce US‐enhanced images (Figures S9 and S10); under the same fundamental frequency, the CEUS image intensity complied with the MI value. Altogether, both the fundamental frequency and MI value could influence the CEUS images of DTTUCA resulting from changes in sonication pressure and vaporisation efficiency. Figure 3C1,C2 shows similar CEUS images that were selected and quantitatively analysed (Figure 3D). According to the Laplace pressure Equation (1), DTTUCA possessed the largest size (Table S1), resulting in the highest CEUS image intensity among all four nanodroplets at each temperature (Figure 3E,F and Tables S4 and S5). PFH‐based DTTUCA nanodroplets had significantly improved boiling point (>70°C), due to which they ensured stable and reversible enhancement under ambient temperature, which was beneficial for improving US imaging and sonography‐guided diagnosis.^10^ Once DTTUCA nanodroplets adhered to the BC cells (Figure 1G), US‐enhanced imaging could be observed in the media (Figure 4D and Figure S19 and Videos S1 and S3) under different MI values.

**FIGURE 3:**
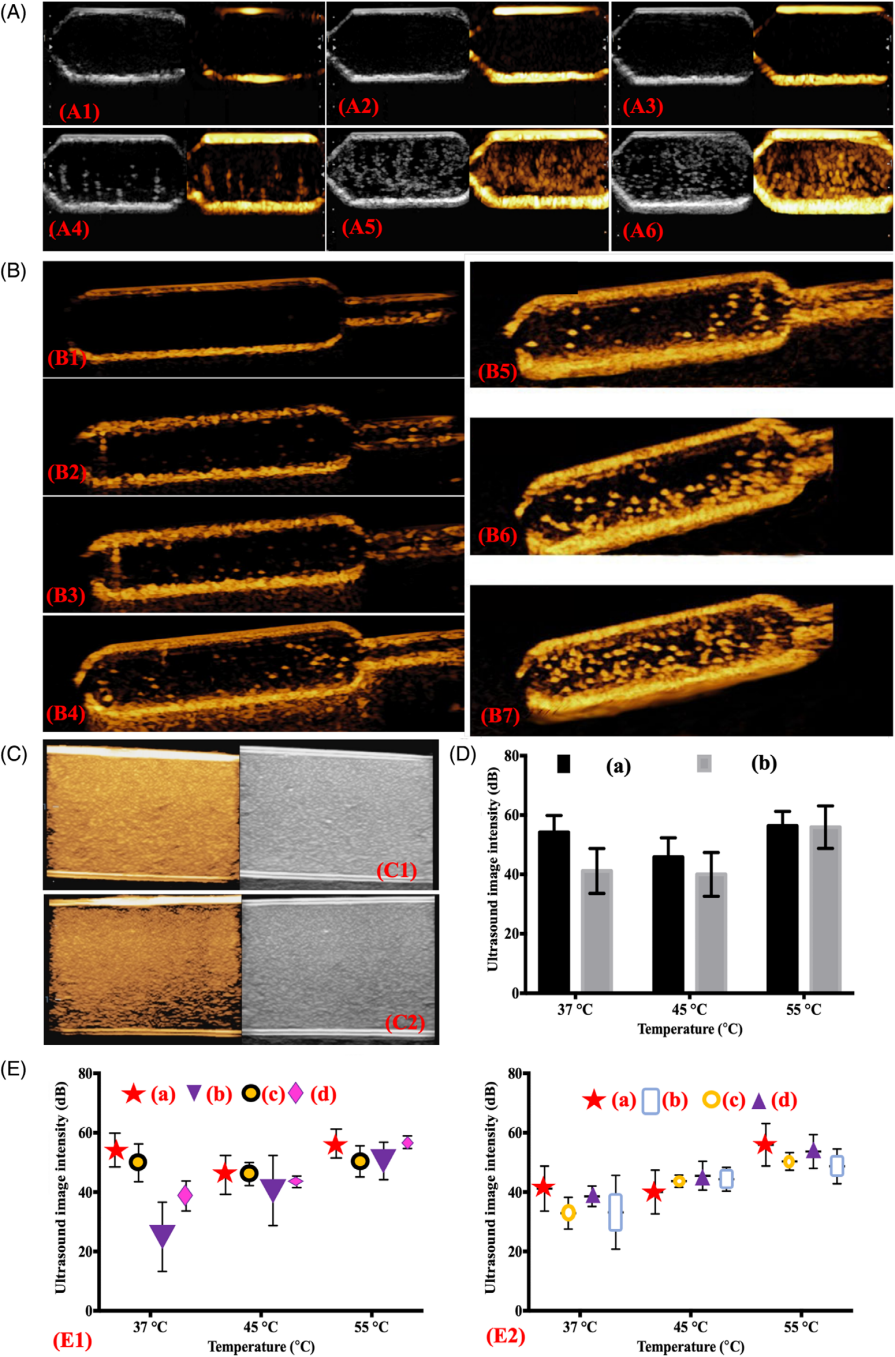
(A) Dual‐targeted theragnostic ultrasound contrast agent (DTTUCA) nanodroplets produced ultrasound (US)‐enhanced images under CEUS and B model. The operational fundamental frequency was 7.5 MHz, and the MI values were 0.2, 0.4, 0.6, 0.8, 1.0, 1.2 (from A1 to A6). (B) DTTUCA nanodroplets promoted US‐enhanced images under CEUS model. The operational fundamental frequency was 13 MHz, and the MI values were 0.08, 0.14, 0.20, 0.30, 0.40, 0.60 and 0.80 (from B1 to B7). (C) DTTUCA nanodroplets produced US‐enhanced images with CEUS and B model. The operational fundamental frequencies were 15 MHz (C1) and 20 MHz (C2), and the MI values were 0.2 (C1) and 0.14 (C2). (D) Quantitative analysis for ROI US images intensity of DTTUCA nanodroplets under different temperatures: fundamental frequencies were 15 MHz (a) and 20 MHz (b), and the MI values were 0.2 (a) and 0.14 (b). (E) Comparison of ROI US images intensity under different temperatures for DTTUCA nanodroplets (a), untargeted mPEG_2k_‐PCL nanodroplets (b), cRGD‐PEG_2k_‐PCL nanodroplets (c) and untargeted mPEG_2k_‐PCL@DOX nanodroplets (d). The fundamental frequencies were 15 MHz (E1) and 20 MHz (E2), and the MI values were 0.21 (E1) and 0.14 (E2)

**FIGURE 4:**
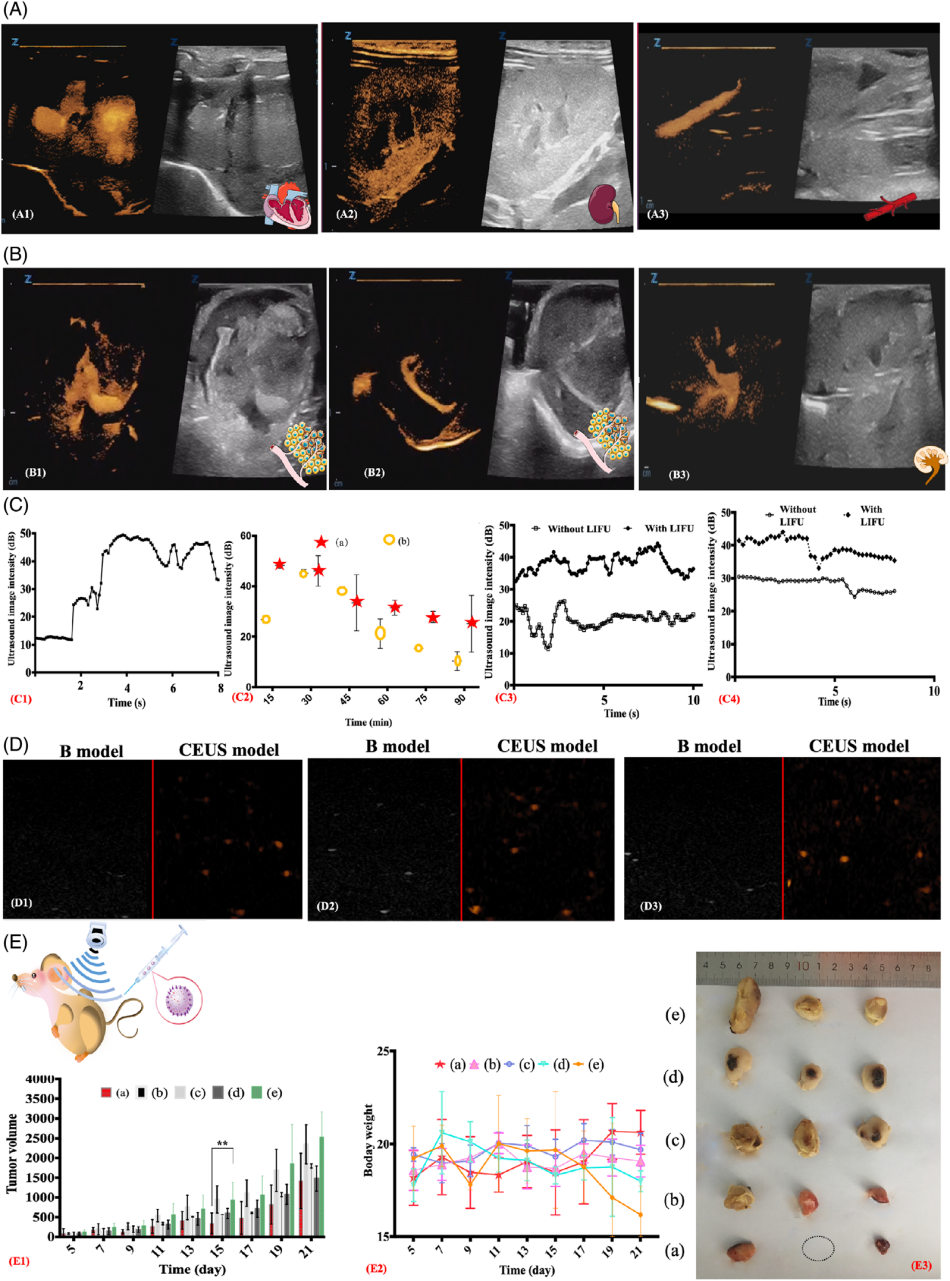
(A) Dual‐targeted theragnostic ultrasound contrast agent (DTTUCA) nanodroplets induced ultrasound (US)‐enhanced images of SD rats under CEUS and B model, from A1 to A3 were heart, kidney and aorta abdominalia tissues. The CEUS images were obtained by intravenous injection via tail. (B) DTTUCA induced US‐enhanced images of the tumour‐bearing nude mice; B1 and B2 showed details of nude mice tumour via in situ injection, B3 showed US‐enhanced images for nude mice kidney via intravenous injection. The fundamental frequency and MI were 15 MHz and 0.21 for both A and B. (C) Quantitative analysis for the CEUS images of DTTUCA in vivo: variations of the US imaging intensity in tumour while DTTUCA nanodroplets were injected in situ within 10 s (C1); US image intensity in tumour (a) and kidney (b) of nude mice (C2); variations of the US imaging intensity in tumour (C3) and kidney (C4) at the preset time point (30 min after the intravenous injection) with and without the 2‐min LIFU radiation. (D) US‐enhanced images of the MCF‐7 cells de‐bubbles medium, MCF‐7 cells were co‐incubated with DTTUCA nanodroplets for 40 min in an ice bath, then DTTUCA were removed, triple washed and replaced by de‐bubbles medium, and observed under CEUS and B model. The fundamental frequency was 7.5 MHz and MI were 0.8 (D1), 1.0 (D2) and 1.2 (D3), respectively. (E) Results of anticancer effects on 4T1 tumour‐bearing nude mice: variations of nude mice weight (E1) (excluded the tumour weight, *n* = 6) and tumour volume (E2) (*n* = 6) for all the five groups (a–e). Anatomical tumour pictures for the same five groups (E3). Group (a) was DTTUCA with LIFU irradiation, group (b, c, d and e) was DTTUCA, mPEG_2k_‐PCL@DOX nanodroplets, DOX•HCl and saline, respectively, and group (a, b, c and d) had the same equivalent weight DOX; **p < .01

The DTTUCA nanodroplets were expected to improve the ultrasonic diagnostic image quality using the CEUS model in vivo. In SD rats, noteworthy enhancements were seen in US sagittal images of the heart, kidney, abdominal aorta and liver (Figure 4A and Figures S11–S13). In nude mice, distinct renal shape and tumour details, such as cavities and major and tiny vessels, could be seen in CEUS images (Figure 4B and Figure S14), besides the regular enhancement and extinction (Figures S15–S17). As shown in Figure 4C1, the tumour US image intensity in nude mice increased from 12.3 ± 0.3 to 48.0 ± 0.9 dB within 2 s. The US image enhanced intensity maintained for a long time (Figure S18), even 30 min later, it was still 45.1 ± 1.4 dB (Figure 4C2). LIFU irradiation along with DTTUCA could trigger an additional enhancement of the acoustic intensity in CEUS imaging (Figure 4C). Altogether, DTTUCA exhibited significant CEUS enhancement in vivo. Together with LIFU irradiation, DTTUCA substantially suppressed tumour growth in nude mice (Figure 4E and Figure S19). DTTUCA and LIFU together resulted in the highest bodyweight and lowest tumour volume, indicating minimum adverse reactions and the best therapeutic outcome. Dissectional analysis and anatomical photography also confirmed that with LIFU exposure, DTTUCA could effectively release drugs and achieve excellent antitumour efficacy. Altogether, as a targeted therapeutic agent, DTTUCA demonstrated appreciable CEUS imaging and antitumour efficacy.

DTTUCA exhibited enhanced US imaging, enhanced cellular uptake and increased cytotoxicity against BC cells in vitro. With LIFU exposure, DTTUCA could markedly inhibit BC growth in vivo. Therefore, the newly explored dual‐targeted nanodroplets show great potential in targeted theragnostic for BC.

## CONFLICT OF INTEREST

The authors declare that there is no conflict of interest.

## Supporting information



Supporting Information

Supporting Information
