# Supplementary material for: Preparation and evaluation of ultrasound‐mediated dual‐targeted theragnostic systems utilising phase‐changeable polymeric nanodroplets on the integrin ανβ3 overexpressed breast cancer
Source: Clin Transl Med. 2021 Oct 14;11(10):e607. doi: 10.1002/ctm2.607 (PMC8516363; doi:10.1002/ctm2.607)
Supplement: Supplementary file 1 — Supporting Information [file CTM2-11-e607-s001.pdf]

Supporting information

Preparation and evaluation of ultrasound-mediated dual-targeted theragnostic systems utilizing phase-changeable polymeric nanodroplets on the integrin $\alpha_v\beta_3$ over-expressed breast cancer

Running head: Polymeric nanodroplets in theragnostics

Na Li[a,b,#], Shaobo Duan[a,#], Yiwei Wang[a], Linlin Zhang[a], Yongqing Chen[a], Juan Zhang[a], Ruiqing Liu[a], Yaqiong Li[a], Luwen Liu[a], Shanshan Ren[a], Ye Zhang[a], Yuqi Guo[a,c], Zhenyu Ji[*b], Lianzhong Zhang[*a]

[a] Henan Provincial People's Hospital; People's Hospital of Henan University; People's Hospital of Zhengzhou University, Zhengzhou 450003, PR China

[b] Institute of Medical and Pharmaceutical Sciences, Zhengzhou University, Zhengzhou 450003, PR China

[c] Henan International Joint Laboratory for Gynecological Oncology and Nanomedicine, Henan Provincial People's Hospital; People's Hospital of Zhengzhou University, Zhengzhou 450003, PR China

# These two authors contribute equally to this manuscript.

**Correspondence to*: Lianzhong Zhang. Henan Provincial Engineering Technology Research Center of Ultrasonic Molecular Imaging and Nanotechnology, Zhengzhou University People's Hospital; Henan Provincial People's Hospital, Zhengzhou, Henan, 450003, China. E-mail: zlz8777@163.com. Zhenyu Ji, Institute of Medical and Pharmaceutical Sciences, Zhengzhou University, Zhengzhou 450003, PR China, E-mail address: jizhenyu@zzu.edu.cn;

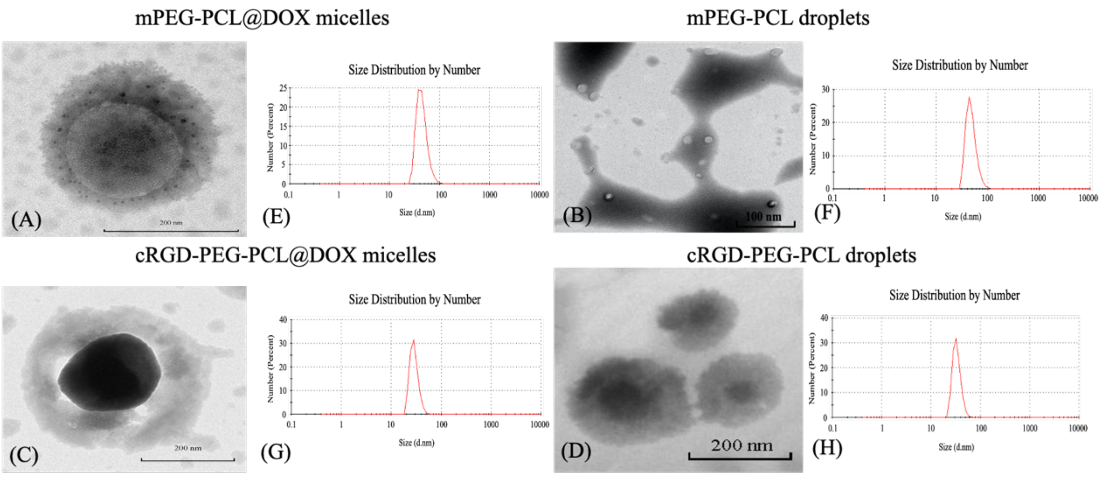

Fig. S1 Morphology and size distribution studies: TEM (from A to D) and DLS (from E to H) results for mPEG$_{2k}$-PCL@DOX micelles, mPEG$_{2k}$-PCL nanodroplets, cRGD-PEG$_{2k}$-PCL@DOX micelles, and cRGD-PEG$_{2k}$-PCL nanodroplets, respectively.

All of the nanoparticles, mPEG$_{2k}$-PCL@DOX micelles, mPEG$_{2k}$-PCL nanodroplets, cRGD-PEG$_{2k}$-PCL@DOX micelles, and cRGD-PEG$_{2k}$-PCL nanodroplets, were sphere shape and had a uniform size distribution.

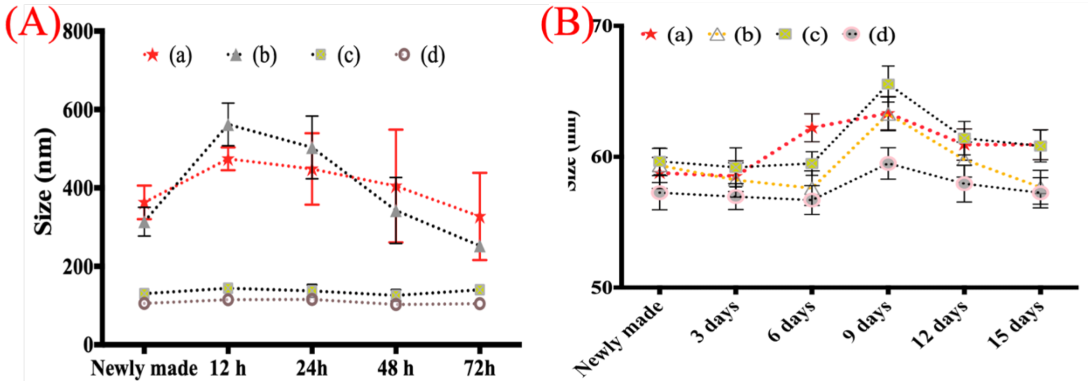

Fig. S2 Stability studies: (A) Variations of DLS particle size for DTTUCA (a), cRGD-PEG$_{2k}$-PCL nanodroplets (b), mPEG$_{2k}$-PCL@DOX nanodroplets (c) and mPEG$_{2k}$-PCL nanodroplets (d) within 72 h (n = 3); (B) Variations of DLS particle size for cRGD-PEG$_{2k}$-PCL @DOX nanomicelles (a), cRGD-PEG$_{2k}$-PCL nanomicelles (b), mPEG$_{2k}$-PCL@DOX nanomicelles (c) and mPEG$_{2k}$-PCL nanomicelles (d) within 15

days (n = 3).

DTTUCA nanodroplets, cRGD-PEG$_{2k}$-PCL nanodroplets, mPEG$_{2k}$-PCL@DOX nanodroplets and mPEG$_{2k}$-PCL nanodroplet could keep their shape and size within 72 h, while cRGD-PEG$_{2k}$-PCL @DOX nanomicelles, cRGD-PEG$_{2k}$-PCL nanomicelles, mPEG$_{2k}$-PCL@DOX nanomicelles and mPEG$_{2k}$-PCL nanomicelles could maintain their shape and size within 15 days.

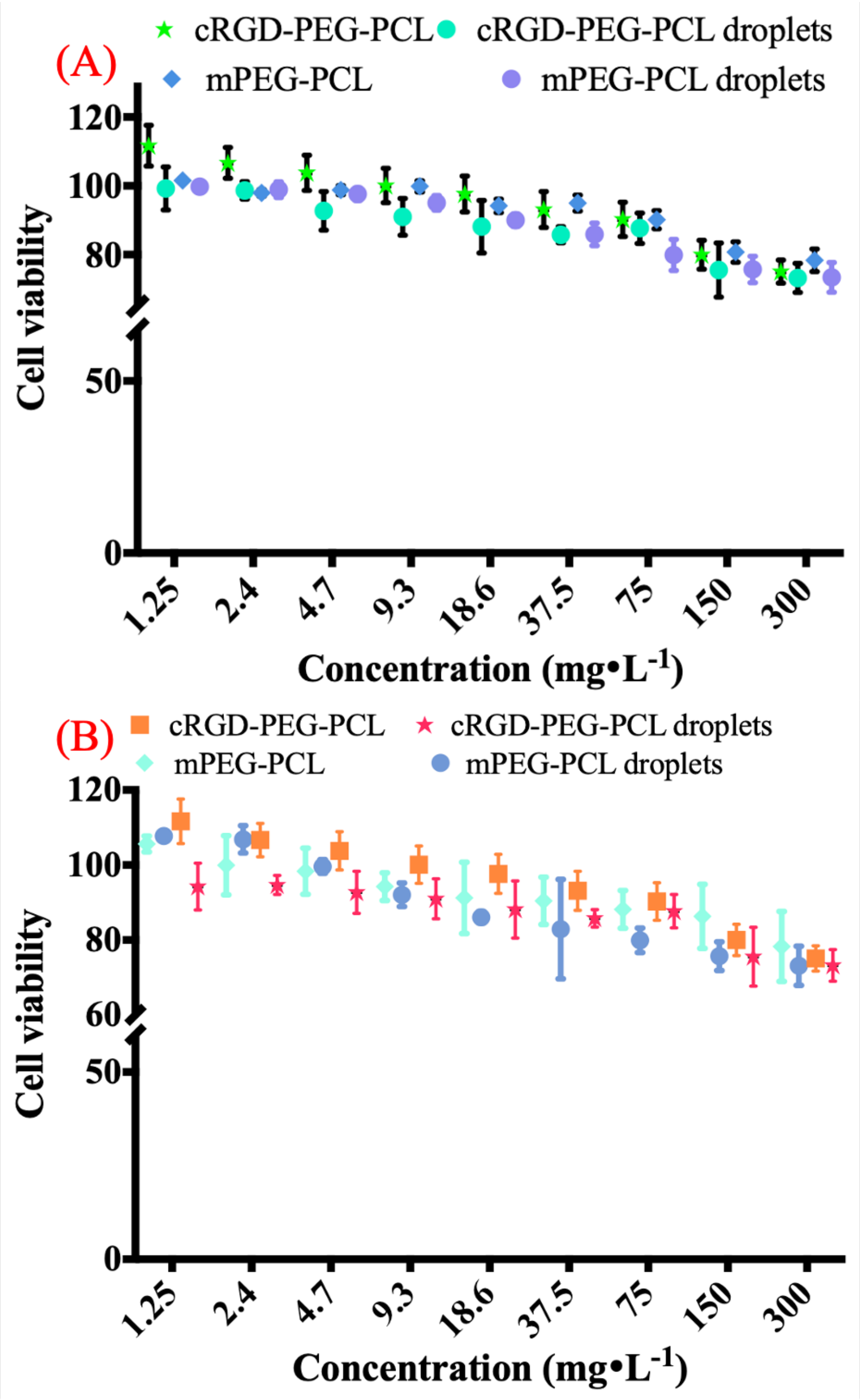

Fig. S3 Cell viabilities of polymeric micelles and nanodroplets against MCF-7 cells (A) and 4T1 cells (B) which did not contain DOX.

Without chemotherapeutic DOX, polymeric micelles and nanodroplets showed little harm to MCF-7 cells and 4T1 cells.

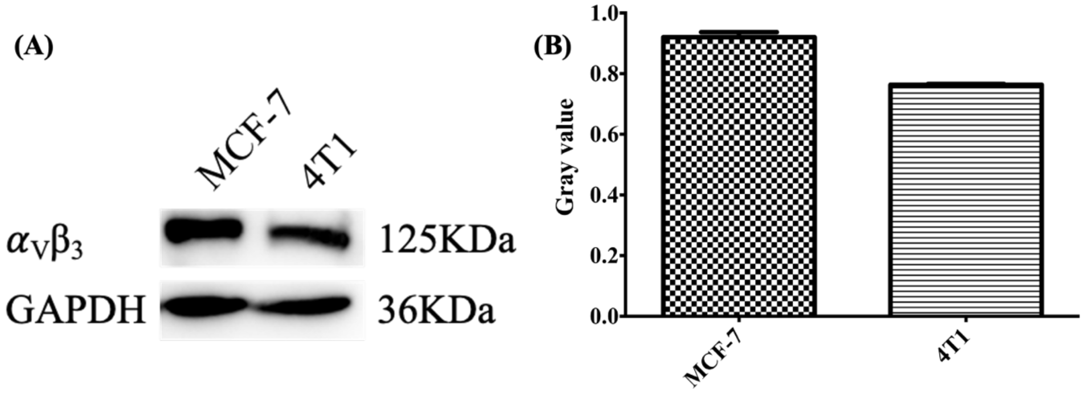

Fig. S4 The qualitative and quantitative analysis of $\alpha_V\beta_3$ integrins on MCF-7 and 4T1 cells.

The $\alpha_V\beta_3$ integrins was significant overexpressed on both MCF-7 and 4T1 cells.

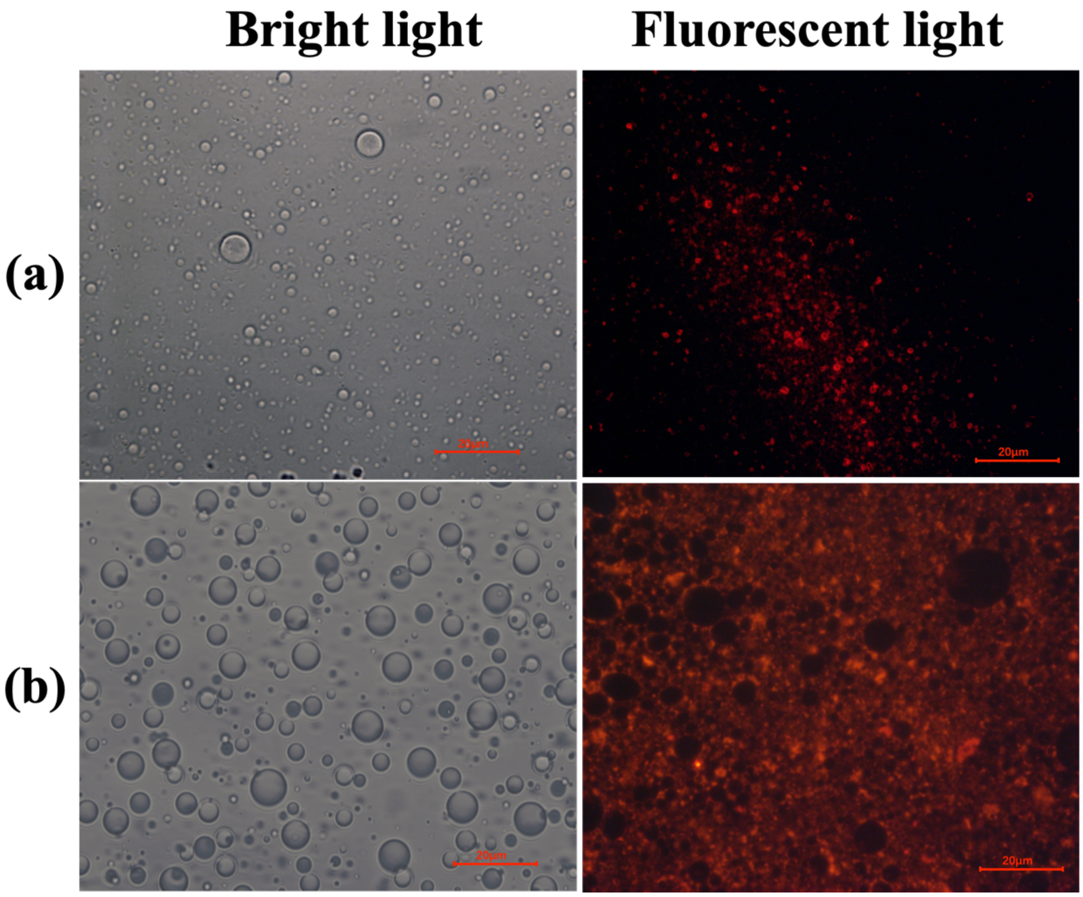

Fig. S5 Representative microphotographs of DTTUCA nanodroplets with different power level of LIFU exposures for 15s, (a) LIFU1 0.5 W/cm^2, and (b) LIFU4 3.5 W/cm^2.

With LIFU irradiation, DTTUCA nanodroplets would display fast vaporization, converting into micro size bubble, and the red fluorescent DOX gathered around the polymeric shells, PFH distributed in the center which was blank dark in the fluorescent pictures. Comparing (a) LIFU1 (0.5 W/cm^2) with (b) LIFU4 (3.5 W/cm^2), while increasing the ultrasonic power the bubble diameter would increase rapidly too.

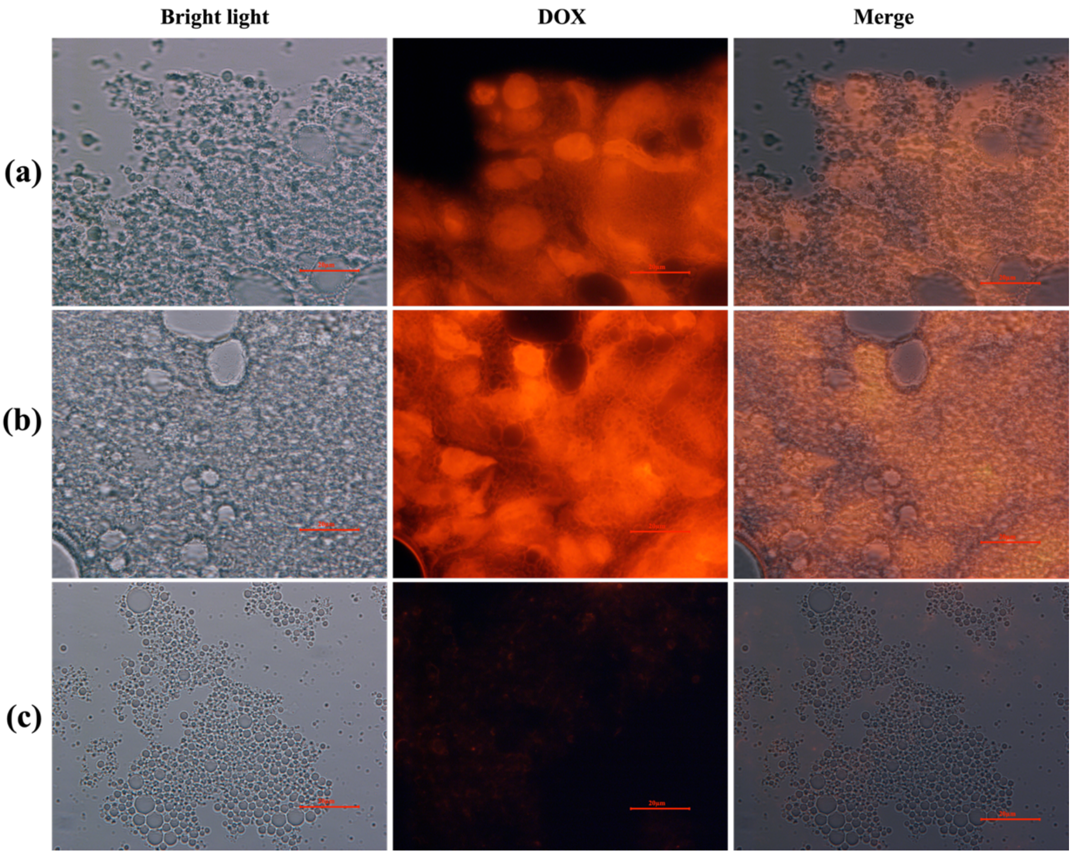

Fig. S6 Representative microphotographs of MCF-7 cells treated with DTTUCA nanodroplets for 40 min: group (a) without LIFU exposures; group (b) with LIFU1 (0.5 W/cm^2) irradiation for 15s, and group (c) with LIFU2 (1.5 W/cm^2) irradiation for 15s.

With cRGD ligand, DTTUCA nanodroplets could selectively adhere to integrins $\alpha_V\beta_3$-overexpressed MCF-7 cells. While the DTTUCA nanodroplets accumulated on the MCF-7 cells surface, they would undergo a ultrasound stimuli triggered phase shift *in situ*. And the bubble diameter would increase along with the ultrasonic power.

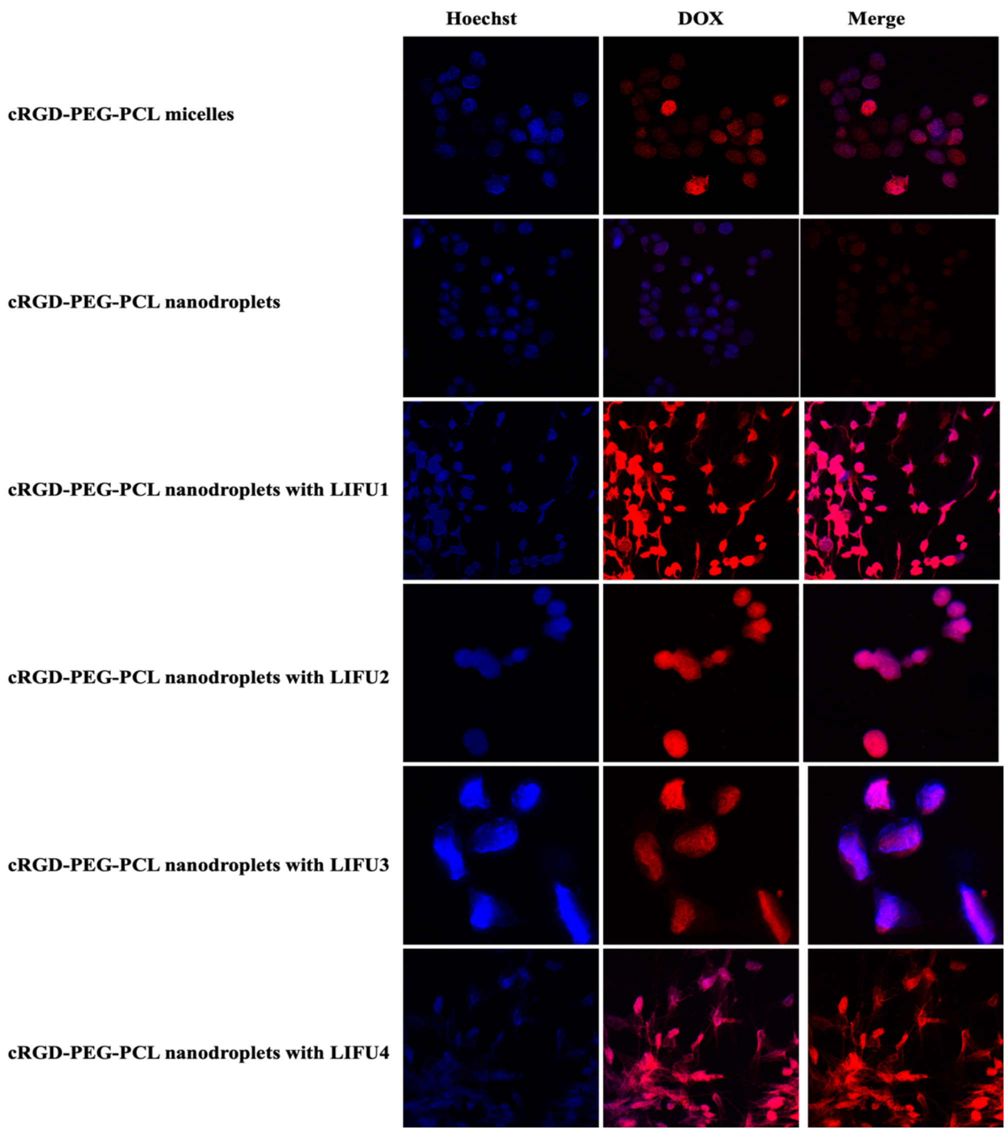

Fig. S7 CLSM of MCF-7 cells that were co-incubation with cRGD-PEF$_{2k}$-PCL@DOX micelles and DTTUCA for 40 min under different levels LIFU exposures for 30 s, LIFU1 0.5 W/cm^2, LIFU2 1.5 W/cm^2, LIFU3 2.5 W/cm^2, and LIFU4 3.5 W/cm^2.

Comparing to cRGD-PEF$_{2k}$-PCL@DOX micelles, DTTUCA nanodroplets with LIFU1 displayed more cellular uptake resulting from the ultrasound-mediated cellular bioeffects. And, the cellular uptake would increase along with the ultrasonic power.

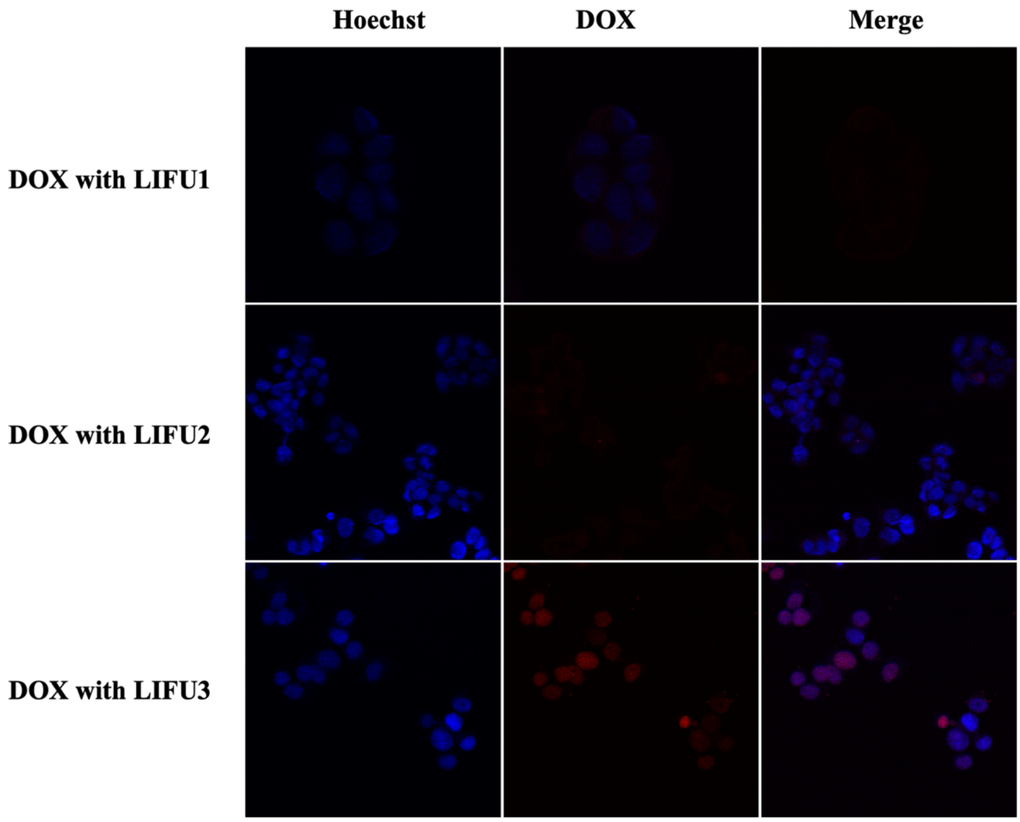

Fig. S8 CLSM of MCF-7 cells treated with DOX•HCl for 40 min under different levels' LIFU exposures for 30s, LIFU1 0.5 W/cm^2, LIFU2 1.5 W/cm^2, and LIFU3 2.5 W/cm^2.

The cellular uptake of DOX•HCl could hardly influent by LIFU irradiation. So, without ultrasound responsive PFH and ultrasonic cavitation, the LIFU irradiation showed limited influence on the cellular uptake of DOX•HCl.

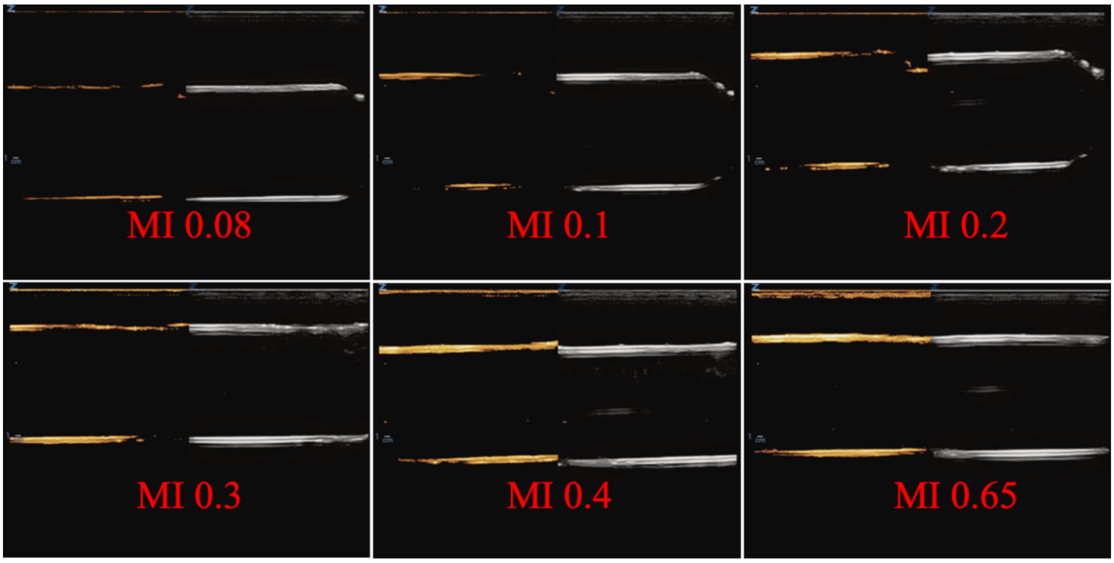

Fig. S9 US images of water under different MI values *in vitro*. Sonography was operated under CEUS and B model whose fundamental frequency was 20 MHz.

No matter what MI value was, there was no enhanced ultrasound imaging in the sonography under CEUS model whose fundamental frequency was 20 MHz.

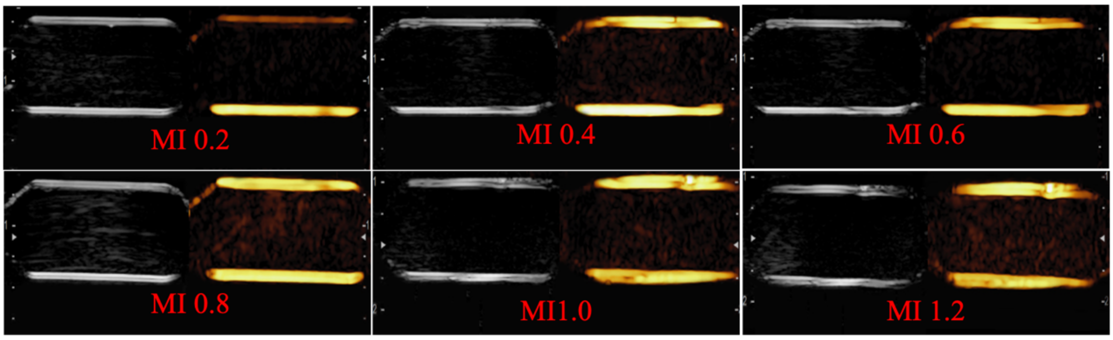

Fig. S10 US images of water under different MI values *in vitro*. Sonography was operated under CEUS and B model with a fundamental frequency of 7.5 MHz.

No matter what MI value was, there was no enhanced ultrasound imaging in the sonography under CEUS model whose fundamental frequency was 7.5 MHz.

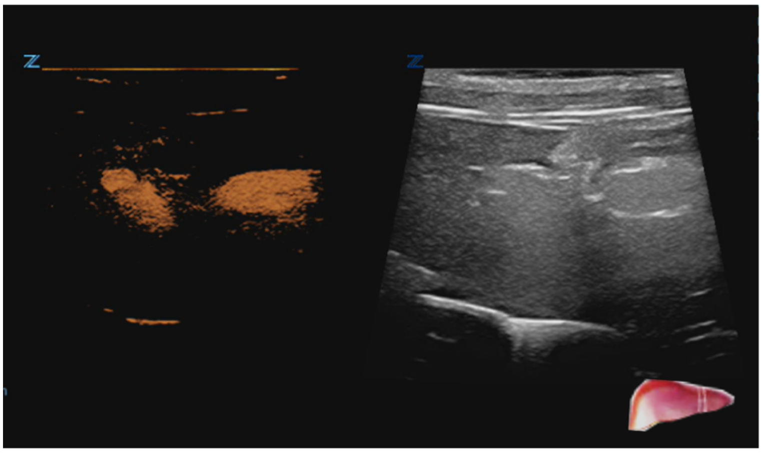

Fig. S11 SD rat liver ultrasound enhanced images induced by DTTUCA nanodroplets under CEUS and B model *via in situ* injection, The fundamental frequency and MI were 15MHz and 0.21.

DTTUCA nanodroplets produced significant enhanced ultrasound imaging in the SD rat liver under CEUS model

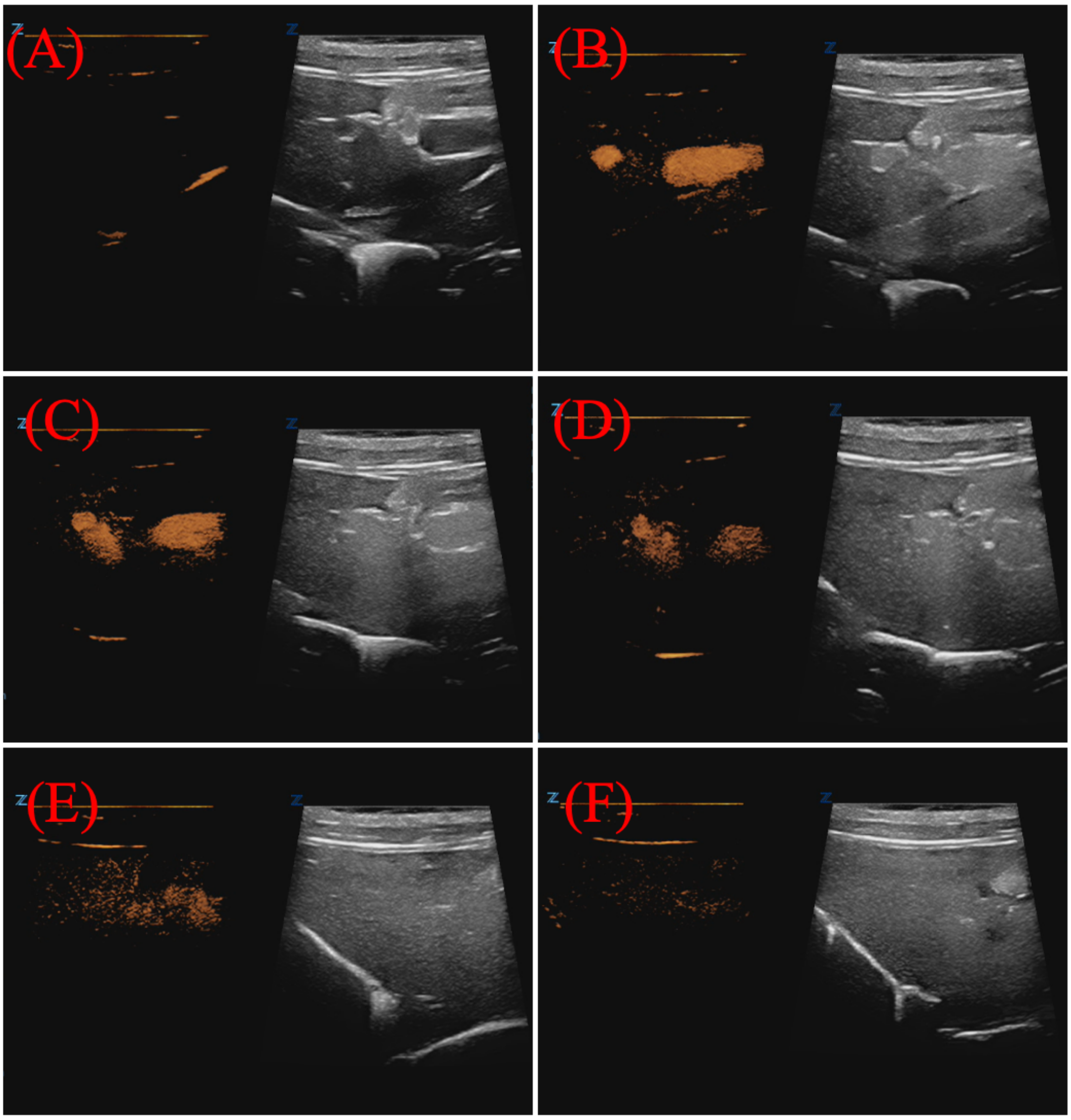

Fig. S12 The enhancement and extinction processes for SD rat liver in sonography induced by DTTUCA nanodroplets under B model and CEUS model.

The US imaging of SD rat liver displayed typical enhancement and extinction processes while the DTTUCA nanodroplets rushed in and left out.

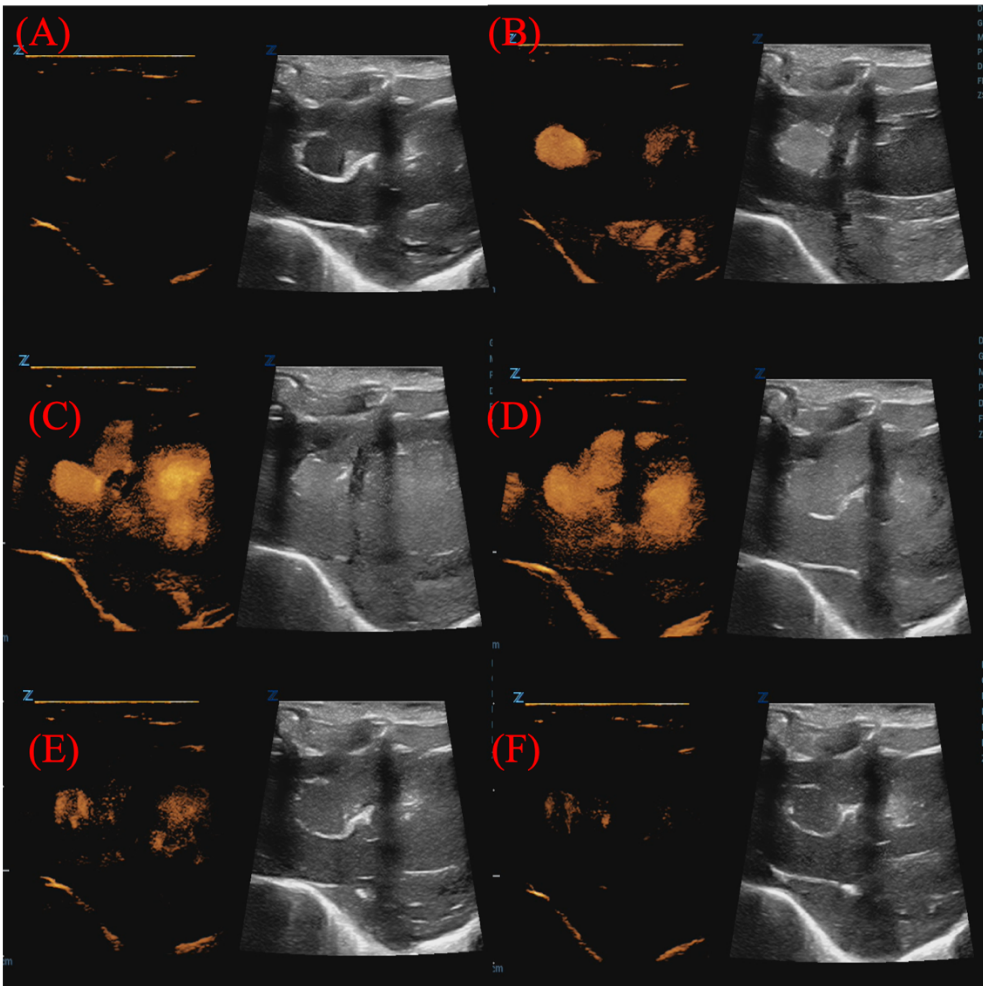

Fig. S13 The enhancement and extinction processes for the SD rat heart in sonography induced by DTTUCA nanodroplets under B model and CEUS model.

The US imaging of SD rat heart displayed typical enhancement and extinction processes while the DTTUCA nanodroplets rushed in and left out.

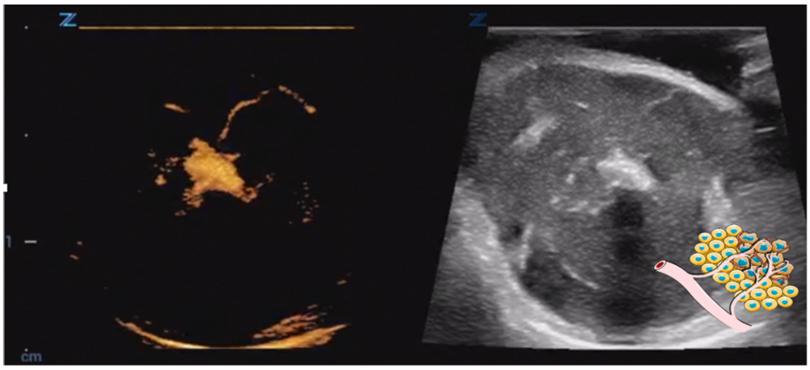

Fig.S14 Ultrasound enhanced images induced by DTTUCA nanodroplets for blood capillary inner nude mice tumor under CEUS and B model by *in situ* injection. The operational fundamental frequency and MI were 15MHz and 0.21.

DTTUCA nanodroplets promoted US enhanced imaging made the inner blood capillary of nude mice tumor clearly to see.

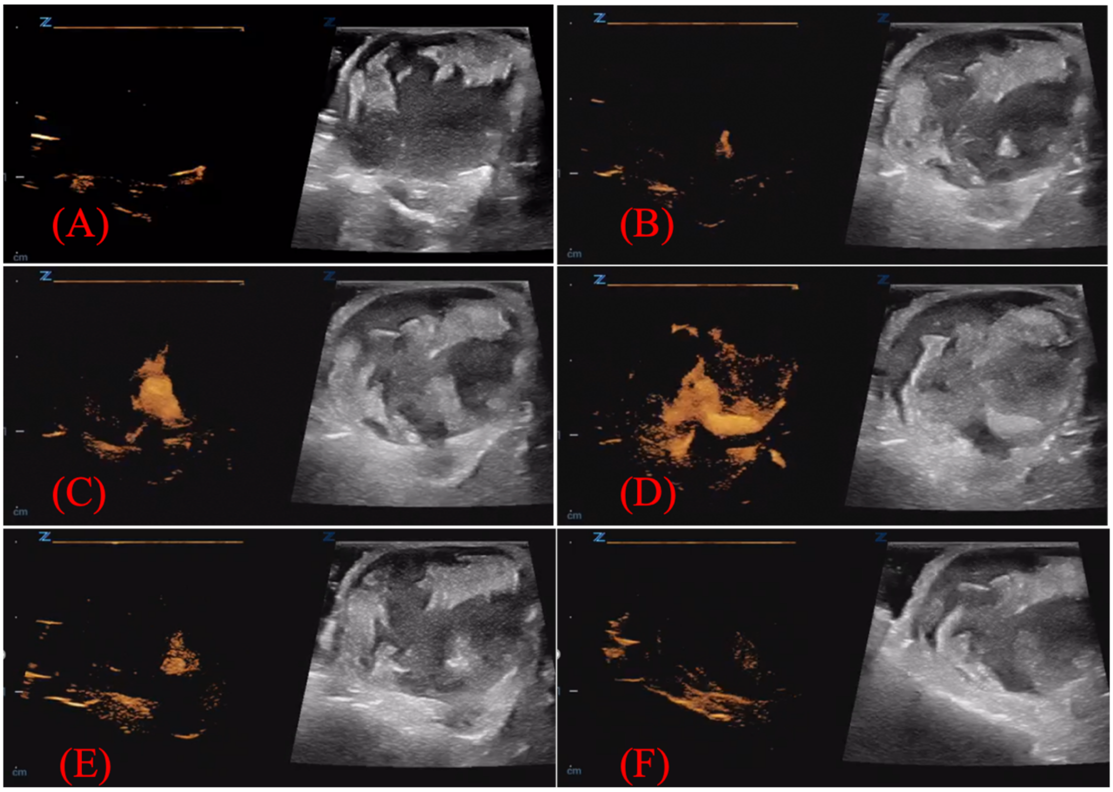

Fig. S15 The enhancement and extinction processes for nude mice tumor in sonography induced by DTTUCA nanodroplets under B model and CEUS model.

The US imaging of nude mice tumor displayed typical enhancement and extinction processes while the DTTUCA nanodroplets rushed in and left out.

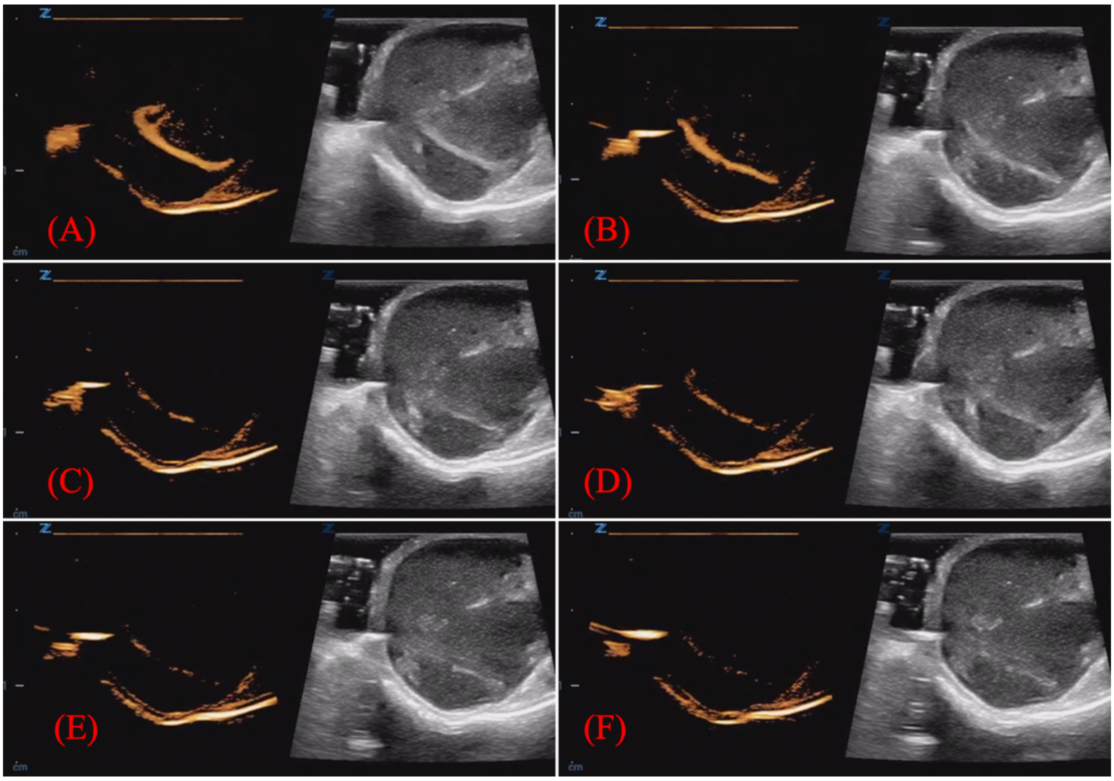

Fig. S16 The enhancement and extinction processes for nude mice main tumor vessel in sonography induced by DTTUCA nanodroplets under B model and CEUS model.

The US imaging of main nude mice tumor vessel displayed typical enhancement and extinction processes while the DTTUCA nanodroplets rushed in and left out along with the blood.

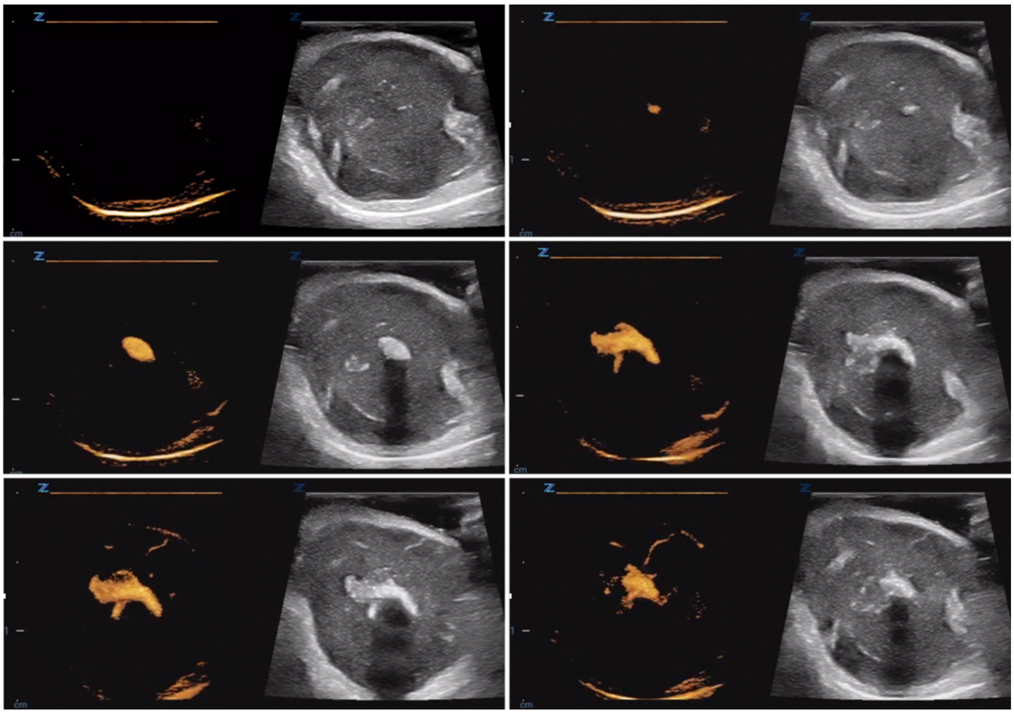

Fig. S17 The DTTUCA enhanced CEUS images *in vivo* for nude mice tinny tumor vessels within 10 second after injection.

The nude mice tinny tumor vessels could be clearly observed in the DTTUCA nanodroplets produced US enhanced images.

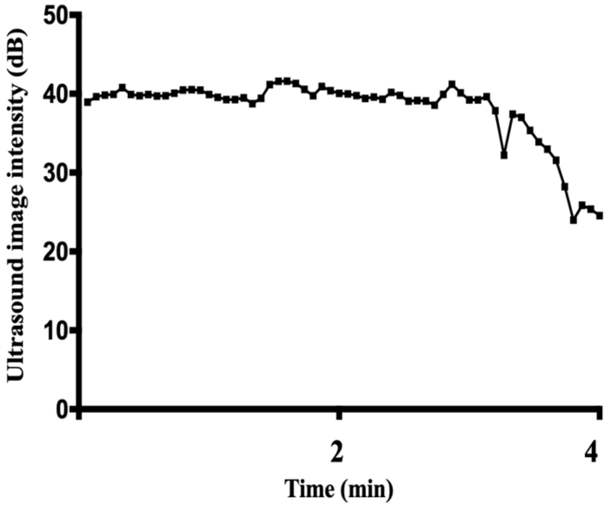

Fig. S18 Variations of the US imaging intensity in nude mice kidney 30 min after the intravenous injection (n = 3).

The increased US imaging intensity in nude mice kidney could maintain for more

than 30 min. And, the US imaging intensity showed little receding in the observed ROI for 2min.

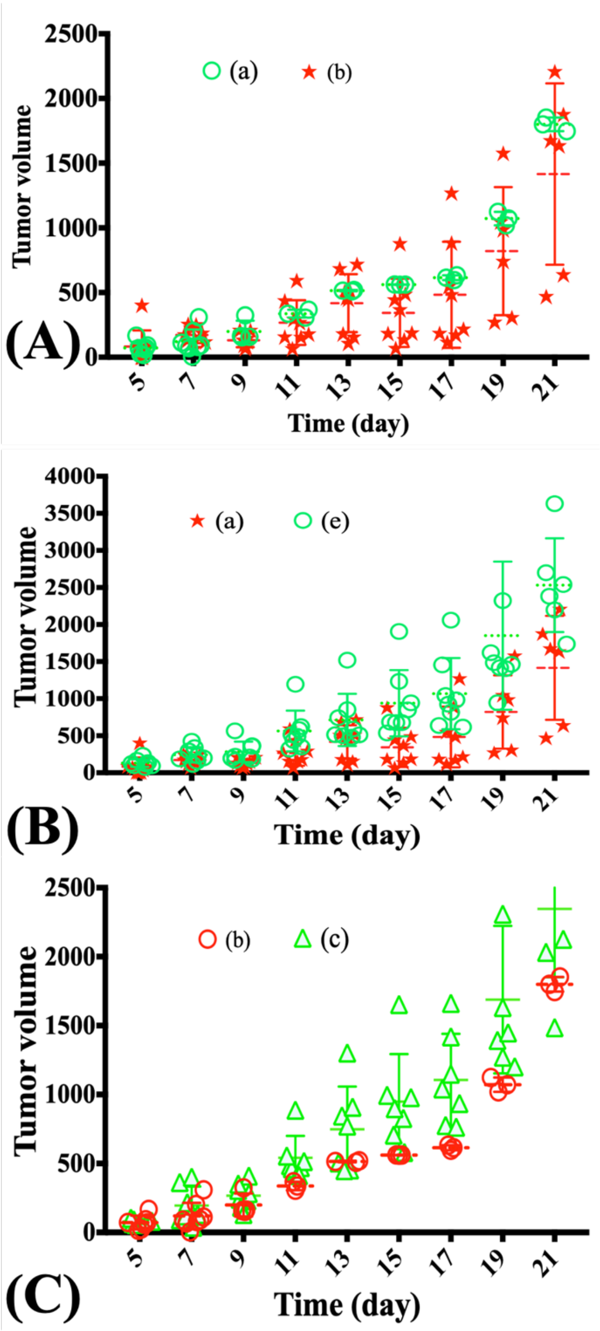

Fig. S19 Comparison of tumor volumes during different groups. Group (a, b, c, d, and e) were DTTUCA with LIFU irradiation (a), DTTUCA (b), mPEG-PCL@DOX nanodroplets (c), DOX•HCl group (d) and saline group (e), respectively. Group (a, b, c,

and d) had the same equivalent weight DOX. n = 6.

Comparing (a) DTTUCA with LIFU irradiation with (b) DTTUCA, LIFU irradiation could help to inhibit the growth of tumor volume, resulting in improved therapeutic effect for DTTUCA nanodroplets (A); Indeed, DTTUCA nanodroplets together with LIFU irradiation could increase antitumor effect and minish side effects (B); cRGD ligand mediated active targeting could help to improve the therapeutic effect of nanodroplets (C).

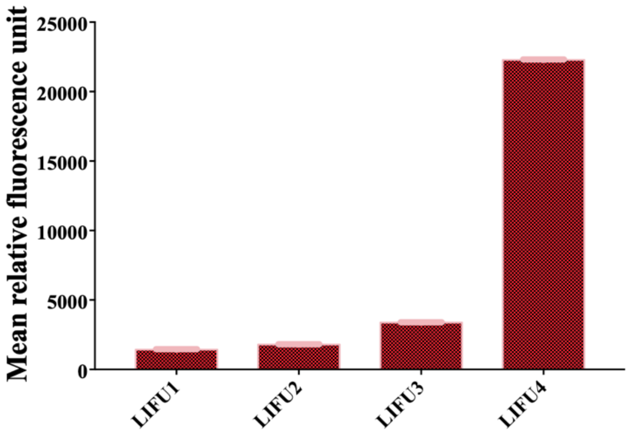

Fig. S20 FCM results *in vitro*: mean relative fluorescence intensities of the cRGD-PEG$_{2k}$-PCL nanodroplets with LIFU1, LIFU2, LIFU3 and LIFU4 irradiation against MCF-7 cells; LIFU1, LIFU2, LIFU3, and LIFU4 means the low-intensity focused ultrasound whose acoustic intensities were of 0.5 W/cm^2, 1.5 W/cm^2, 2.5 W/cm^2, and 3.5 W/cm^2, respectively.

LIFU stimuli can obviously improve the cellular uptake of DTUUCA. The FL intensity became progressively strong from LIFU 1 to LIFU4.

Table. S1 Size distribution of DTTUCA nanodroplets, $mPEG_{2k}$-PCL@DOX nanodroplets, $mPEG_{2k}$-PCL micelles and cRGD-PEG_{2k}-PCL micelles measured by dynamic light scattering.

DTTUCA		$mPEG_{2k}$-PCL@DOX nanodroplets		$mPEG_{2k}$-PCL micelles		cRGD-PEG_{2k}-PCL micelles	
Diameter (nm)	SD	Diameter (nm)	SD	Diameter (nm)	SD	Diameter (nm)	SD
363.1	43.0	130.37	11.8	57.25	1.3	59.33	1.3

Table. S2 The FCM results of cRGD-PEG_{2k}-PCL group (a), $mPEG_{2k}$-PCL@DOX group (b) and DOX•HCl groups (c) under different conditions against MCF-7 cells.

	cRGD-PEG_{2k}-PCL Groups(a)		$mPEG_{2k}$-PCL Groups (b)		DOX·HCl Groups (c)	
	Mean FL intensity	SD	Mean FL intensity	SD	Mean FL intensity	SD
Nanomicelles	2817	12	574	32	643	28
Nanodroplets	1793	20	1013	28	/	/
Nanodroplets with LIFU3[1]	3374	23	551	27	185	20
Nanodroplets with LIFU4[2]	22282	32	1293	40	109	23

[1] LIFU3 means the acoustic intensity of the low-intensity focused ultrasound was 2.5 W/cm^2.

[2] LIFU4 means the acoustic intensity of the low-intensity focused ultrasound was 3.5 W/cm^2.

Table. S3 FCM results of cRGD-PEG_{2k}-PCL group (a), $mPEG_{2k}$-PCL@DOX group (b) and DOX•HCl groups (c) under different conditions against 4T1 cells.

	cRGD-PEG_{2k}-PCL Groups (a)		$mPEG_{2k}$-PCL Groups (b)		DOX·HCl Groups (c)	
	Mean FL intensity	SD	Mean FL intensity	SD	Mean FL intensity	SD
Nanomicelles	1568	13	1032	23	707	41
Nanodroplets	1576	32	1407	27		
Nanodroplets with LIFU3[1]	1797	15	940	19	626	27
Nanodroplets with LIFU4[2]	1843	21	607	34	427	38

[1] LIFU3 means the acoustic intensity of the low-intensity focused ultrasound was 2.5 W/cm^2.

[2] LIFU4 means the acoustic intensity of the low-intensity focused ultrasound was 3.5 W/cm^2.

Table. S4 Mean CEUS intensity of DTTUCA nanodroplets, cRGD-PEG_{2k}-PCL nanodroplets, $mPEG_{2k}$-PCL nanodroplets, and $mPEG_{2k}$-PCL@DOX nanodroplets at 37 °C, 45 °C and 55 °C. MI and fundamental frequency were 0. 21 and 15 MHz, respectively.

	DTTUCA		cRGD-PEG_{2k}-PCL		$mPEG_{2k}$-PCL@DOX		$mPEG_{2k}$-PCL	
	Mean[1] (dB)	SD[2]	Mean (dB)	SD	Mean (dB)	SD	Mean (dB)	SD
37 °C	54.2	5.7	49.9	6.4	38.7	5.0	24.9	11.7
45 °C	45.8	6.5	46.1	3.9	43.4	2.0	40.5	11.8
55 °C	56.4	4.9	50.3	5.2	56.8	2.1	50.5	6.3

[1] Mean is a representation of Mean CEUS imaging intensity.

[2] SD is a representation of STDEV i.e., standard deviation.

Table. S5 Mean CEUS intensity of DTTUCA nanodroplets, cRGD-PEG$_{2k}$-PCL nanodroplets, mPEG$_{2k}$-PCL nanodroplets, and mPEG$_{2k}$-PCL@DOX nanodroplets at 37 °C, 45 °C and 55 °C. MI and fundamental frequency were 0.14 and 20 MHz respectively. Loading with chemotherapeutic DOX, DTTUCA and mPEG$_{2k}$-PCL@DOX nanodroplets displayed a bit of expanding compared with cRGD-PEG$_{2k}$-PCL and mPEG$_{2k}$-PCL nanodroplets, respectively.

	DTTUCA		cRGD-PEG$_{2k}$-PCL		mPEG$_{2k}$-PCL@DOX		mPEG$_{2k}$-PCL	
	Mean[1] (dB)	SD[2]	Mean (dB)	SD	Mean (dB)	SD	Mean (dB)	SD
37 °C	41.2	7.6	32.9	5.3	38.6	3.5	33.2	12.4
45 °C	40.0	7.4	43.7	2.0	45.5	4.8	44.3	4.0
55 °C	55.9	7.2	50.3	3.0	53.7	5.7	48.7	5.8

[1] Mean is a representation of Mean CEUS imaging intensity.

[2] SD is a representation of STDEV i.e., standard deviation.

Table. S6 Mean CEUS intensity of the MCF-7 cells with de-bubbles medium, MCF-7 cells were co-incubated with DTTUCA nanodroplets for 40 min in an ice bath, then DTTUCA were removed, triple washed and replaced by de-bubbles medium, and the de-bubbles medium were observed under CEUS and B model. The operational fundamental frequency was 7.5 MHz, and MI was 0.8, 1.0 and 1.2, respectively. The ECHO intensity of ROI under CEUS model was analyzed and listed as follows:

MI	ECHO intensity ranges (dB)
0.8	85~102
1.0	82~110
1.2	133~184

Materials and Experiments

1. Materials and Measurements

Doxorubicin (DOX) was purchased from Meilunbio® (Dalian Meilun Biotechnology Co., Ltd, Dalian, China), Cyclo (Arg-Gly-Asp-d-Tyr-Lys) peptides (cRGD) modified poly(ethyleneglycol)-poly(caprolactone) (cRGD-PEG-PCL) was purchased from Xi'an Ruixi Biotech Inc. (Xi'an, China). Dialysis bag (MWCO 2000 Da) was purchased from Vita Chemical Reagent Co., Ltd. (Shanghai, China). Acetone was purchased from Sigma-Aldrich. Hoechst33342 and cell counting kit-8 (cck-8) were purchased from Beyotime (Shanghai, China). Other reagents were used as received. The cellular uptake was examined employing a confocal laser scanning microscopy (CLSM, Leica TCs SP5) and Flow cytometry (BD, FACSCalibur, USA). The shape and size of the cancer nanomedicines were measured by transmission electron microscopy (TEM) and dynamic light scattering (DLS), respectively. The cell viability was determined by microplate reader (Bio-Tek, USA).

2. Preparation of DTTUCA and other Relevant Nanoparticles

DTTUCA namely cRGD-PEG$_{2k}$-PCL@DOX nanodroplets and the other relevant nanoparticles i.e. cRGD-PEG$_{2k}$-PCL micelles, cRGD-PEG$_{2k}$-PCL nanodroplets, mPEG$_{2k}$-PCL micelles, mPEG$_{2k}$-PCL nanodroplets, and mPEG$_{2k}$-PCL@DOX nanodroplets were produced by solvent evaporation method and ultrasonic emulsification [37]. In detail, a small amount of cRGD-PEG$_{2k}$-PCL (25 mg) was dissolved in acetone (2 mL) forming settled solution. Then, ultrapure water (4 mL) was added dropwise into settled solution under stirring, then, acetone was evaporated by a rotary vacuum evaporator until the settled solution changed to creamy white colloidal solution forming cRGD-PEG$_{2k}$-PCL nanomicelle. Therefore, the PFC (2% in volume) was introduced to the newly made cRGD-

PEG_{2k}-PCL nanomicelle quickly which was emulsified in ice-water for 5 min utilizing a 20-kHz ultrasonic processor (SCIENTZ08-2, SCIENTZ, China) forming cRGD-PEG_{2k}-PCL nanodroplets. Meanwhile, DOX (10 mg) was dissolved in acetone (1 mL) and added into the creamy white colloidal solution dropwise in ice-water while sonicated in 30 min forming red complex mixture. Then, the red mixture was transferred to dialysis tube (MW = 2000, Vita Chemical Reagent Co., Ltd. Shanghai), overnight, and the PFC (perfluorohexan, 2% in volume) was introduced to the red mixture quickly which was emulsified in ice-water for 5 min utilizing a 20-kHz ultrasonic processor (SCIENTZ08-2, SCIENTZ, China) forming cRGD-PEG_{2k}-PCL@DOX nanodroplets. The preparations of $mPEG_{2k}$-PCL micelles, $mPEG_{2k}$-PCL nanodroplets, and $mPEG_{2k}$-PCL@DOX nanodroplets were similar to cRGD-PEG_{2k}-PCL micelles, cRGD-PEG_{2k}-PCL nanodroplets and cRGD-PEG_{2k}-PCL@DOX nanodroplets.

3. Size and Shape Characterization of DTTUCA and the Relevant Nanoparticles

Once the DTTUCA and the relevant nanoparticles were got, they were adjusted to 5 mg/mL and measured by the DLS (Zetasizer Lab) as newly made, and at 12, 24, 48, and 96 h. The nanomicelles were also adjusted to 5 mg/mL and measured by the DLS (Zetasizer Lab) as newly made, and by day 3, 6, 9, 12 and 15. At the same time, all the nanomicelles and nanodroplets were stained and observed on the TEM. In detail, the newly made cRGD-PEG_{2k}-PCL nanomicelles, mPEG-PCL nanomicelles, cRGD-PEG_{2k}-PCL@DOX nanodroplets and $mPEG_{2k}$-PCL@DOX nanodroplets were dripped on the parafilm respectively, the copper screens were dripped into the drop and kept for 10 s. While the copper screens dried, they were dripped into the phosphotungstic acid drops and kept for 10 s. The copper screens were observed by TEM for one night.

4. BC Cells Incubation

MCF-7 cells and 4T1 cells were purchased from Chinese Academy of Sciences (Shanghai, China). All these two cells were cultured in RPMI 1640 medium (Gibco BRL, Paris, France). The cells were supplemented with 10% fetal bovine serum (FBS, HyClone, Logan, UT), streptomycin (100 μg/mL) and penicillin (100 g/mL). MCF-7 cells and 4T1 cells were incubated at 37 ºC with 5% CO_2 atmosphere.

5. Western Blotting (WB) for Detecting the Expression of $a_v\beta_3$/GAPDH

MCF-7 and 4T1 cells were seeded in 96-well plates, respectively, and lysed in buffer containing 2% SDS (Beyotime, Shanghai, China) and quantified by performing a bicinchoninic acid (BCA) assay (TAKARA, AIG1851A). An equal amount of protein was subjected to electrophoresis on SDS–PAGE (Abcam). The $a_v\beta_3$ antibody (Abcam), GAPDH antibody (PROTEINTECH), HRP-labeled Goat Anti-rabbit IgG(H+L) (Beyotime), and HRP-labeled Goat Anti-mouse IgG(H+L) (Beyotime) were operated orderly, and the expression measurement was performed with an enhanced chemiluminescence on Millipore ECL systems.

6. Cellular Uptake of DTTUCA and untargeted Nanoparticles

MCF-7 and 4T1 cells (2×10^6 cells) were seeded and cultured in a 6-well plate for 24 h and further cultured with DOX·HCl, DTTUCA or $mPEG_{2k}$-PCL@DOX nanodroplets. Thirty min later, cells were washed with PBS (pH = 7.4) and stained with Hoechst 33342 (10 mg/L) for 10 min. Images were taken on confocal laser scanning microscope (CLSM) (Leica TCs SP5). The MCF-7 and 4T1 cells were digested, centrifuged, and washed with PBS, respectively, then, all the tree cells were used to analyze the cellular uptake by flow cytometry (FCM) (BD Canto plus, USA).

7. Sonography of the MCF-7 cells medium for molecular imaging study

MCF-7 and 4T1 cells (2 × 10^6 cells) were seeded and cultured in a 6-well plate for 24 h and further cultured with DTTUCA nanodroplets for 40 min in an ice bath, then DTTUCA were removed, triple washed and replaced by de-bubbles medium, and the de-bubbles medium were observed under CEUS and B model. The operational fundamental frequency was 7.5MHz, and MI was 0.8 (D1), 1.0 (D2) and 1.2 (D3), respectively.

8. CCK-8 Test

Cell viability was assessed by CCK-8 agents. MCF-7 and 4T1 cells (8 × 10^3 cells/well) were seeded and cultured in a 96-well plate with 200 μL of culture medium per well for 24 h, respectively. Then, cells were cultured with fresh medium containing different concentrations of DOX·HCl, DTTUCA or PEG_{2k}-PCL@DOX nanodroplets (equivalent DOX·HCl concentration 0.01, 0.02, 0.04, 0.08, 0.16, 0.32, 0.63, 1.25, 2.50, and 5.00 mg/L) for 48 h and culture medium with 10 μL 5 mg/mL CC K-8 for 2 h. Then, the absorbance of each well was measured at 570 nm using an automated BIO-TEK microplate reader (BIotek Synergy H, USA).

9. Sonographic Examination *in vitro*

The newly made DTTUCA nanodroplets were diluted into designed concentration as 1 mg/mL, then they were injected into the plastic pipettes (Shanghai, China) and sealed by clamps for *in vitro* imaging experiments, respectively [38]. Ultrapure water was used as control. The sonography was conducted on a Mindrary ZS3 ultrasound platform (SHENZHEN MINDRAY BIO-MEDICAL ELECTRONICS CO., LTD., Shenzhen, China) which was equipped with a L30 M linear array transducer (5"13 MHz). The US images were acquired under contrast enhanced ultrasound (CEUS) mode with two different detection models with the mechanical indexes (MI) of 0.21 and 0.14, and the corresponding fundamental frequency of 15 and 20 MHz, respectively. Both modes were measured at

37 °C, 45 °C, and 55 °C. All these operations were repeated three times for each MI.

10. Sonographic Examination *in vivo*

Heathy male Sprague-Dawley (SD) rats (ca. 200 g) were randomly divided into A, B, C and D group (6 rats each group). The newly made DTTUCA nanodroplets were injected into (5 mg/kg) tail vein in A, B and C groups to observe aorta abdominalia, heart and kidney, respectively. In group D, the newly made DTTUCA nanodroplets were injected into the liver for *in situ* sonography. A Mindrary ZS3 ultrasound platform with a Mindrary-L30 M transducer was used for the *in vivo* imaging experiments through CEUS mode. The detection frequency and MI used were 15 MHz and 0.21. During the whole measurement, the coupling gel was used to minimize the sound attenuation and acoustic impedance.

Female BALB/c nude mice with xenografted tumor on their right shoulders were randomly divided into E and F groups (6 mice/group). The newly made DTTUCA nanodroplets were injected into (5 mg/kg) tail vein in group E to observe kidney. In group F, the newly made DTTUCA nanodroplets were injected into the tumors for *in situ* sonography. The ultrasound contrast-enhanced imaging for tumor and kidney was observed at the pre-set time points: 0.25, 0.50, 0.75, 1.0, 1.25, and 1.5 h. A single focus was placed at 1.5 or 2.5 cm according to the US images. The imaging target depth was 4 cm. The results were analyzed by the contrast enhanced ultrasound quantification imaging (CEUS-QI) software and tissue tracking quantitative analysis (TTQA) mode.

11. Tumor-Xenografted Mouse Model and Anti-Cancer Treatments

Female BALB/c nude mice (18-20 g) were used to prepare the 4T1 tumor-xenografted mice models for the *in vivo* anticancer activity test. Mice were kept in a light-controlled room at temperature of (23 ± 2) °C and relative humidity of (50 ± 5) %. The experimental procedures were approved by the

Animal Care and Use Committee of the Zhengzhou University People's Hospital, Zhengzhou University. When 4T1 cells reached logarithmic growth phase, they were digested, centrifuged, dispersed in saline (1×10^7 cells/mL), and seeded under their right shoulder of the nude mice. Two days later, all the mice were randomly divided into A, B, C, D and E groups (6 mice/group). The changes of tumor volume and the weight of the nude mice were observed while treated with different conditions. Group A and B were treated with the newly prepared DTTUCA nanodroplets by tail intravenous injection. One hour after injection, group B was treated with low intensity focused ultrasound for 2 min on the tumor areas. Group C, D and E were treated with the newly prepared $mPEG_{2k}$-PCL@DOX nanodropltes, DOX•HCl, and saline (0.9 wt% saline as control), respectively. The dose of the DOX•HCl, DTTUCA and the relevant nanoparticles in all formulations was 5 mg/kg. The formulations were given to nude mice *via* tail intravenous injection by day 5, 7, 9, 11, 13, 15, 17, 19 and 21 after the tumor cell seeding. At the predetermined time points, mice were weighed, and tumor size was measured with a Vernier caliper. The tumor volume was calculated using the following formula:

$$V = a \times b^2/2 \quad \cdots\cdots\cdots\cdots\cdots\cdots\cdots\cdots\cdots\cdots\cdots\cdots\cdots\cdots \quad \text{equation (1)}$$

V was the tumor volume; a was the length of the tumor; b was the width of the tumor.
