# Supplementary figures and images for: Preparation and evaluation of ultrasound‐mediated dual‐targeted theragnostic systems utilising phase‐changeable polymeric nanodroplets on the integrin ανβ3 overexpressed breast cancer
Source: Clin Transl Med. 2021 Oct 14;11(10):e607. doi: 10.1002/ctm2.607 (PMC8516363; doi:10.1002/ctm2.607)

**Video**

**Video-1 MI 0.8**


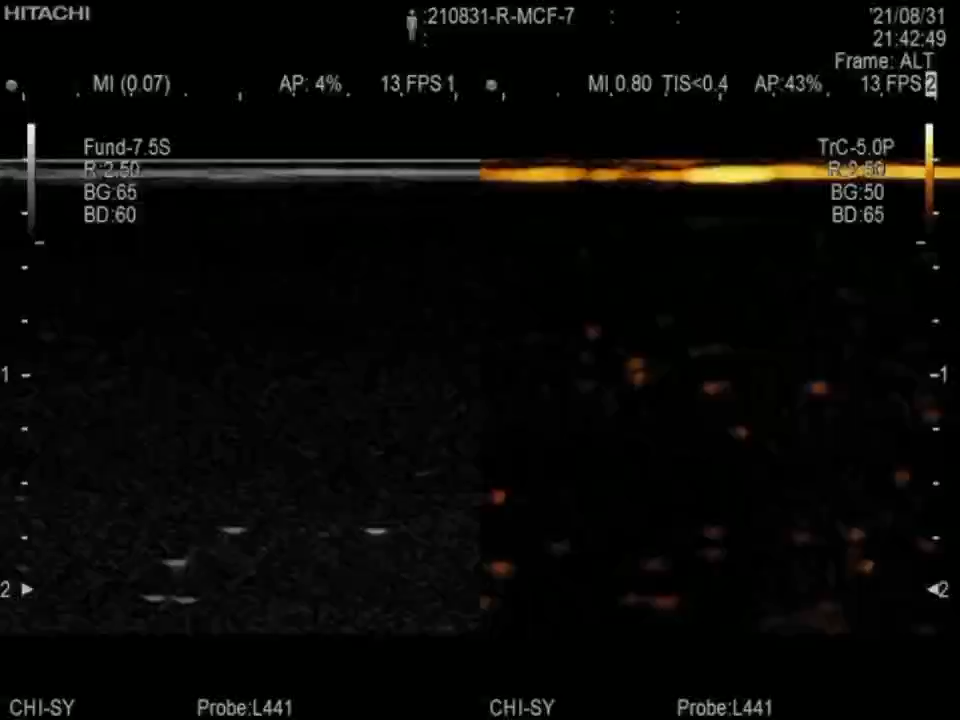


**Video-2 MI 1.0**

**
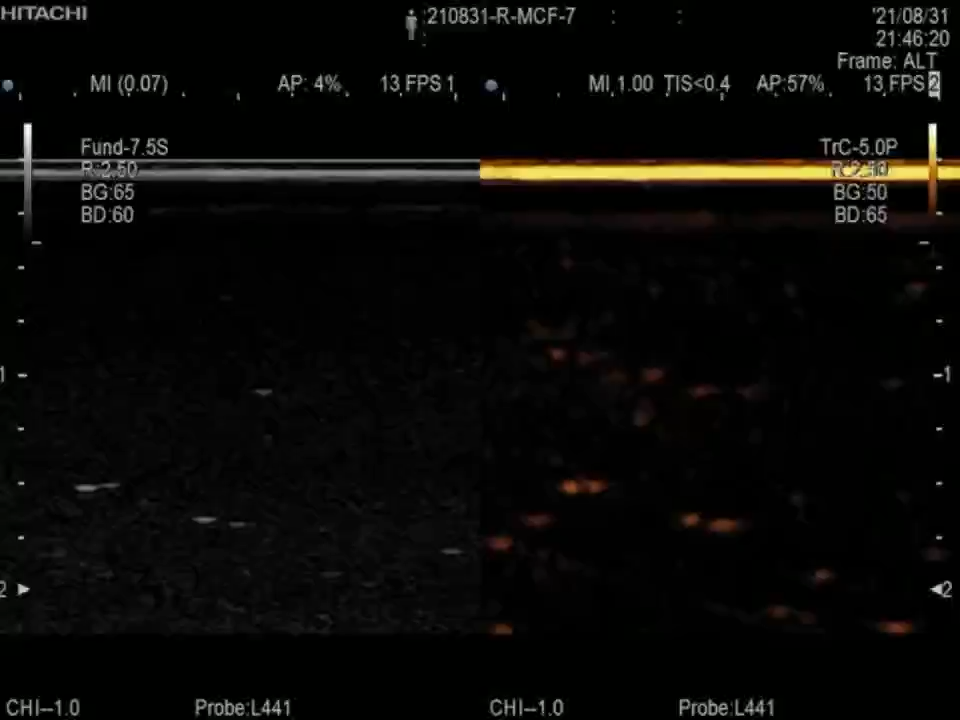
**

**Video-3 MI 1.2**

**
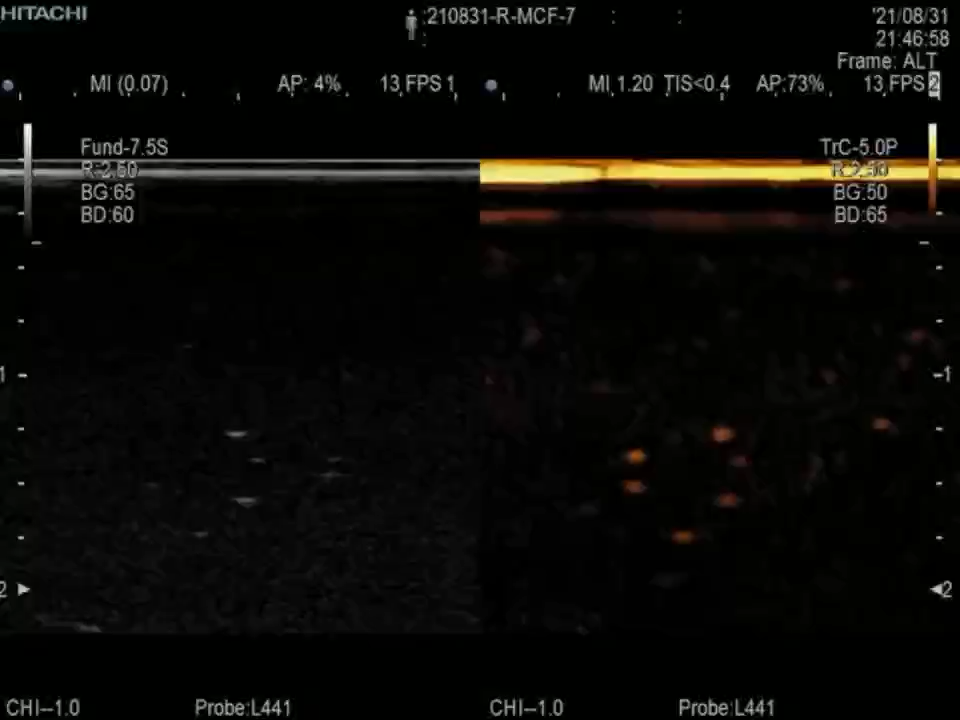
**

Supplement: Supplementary file 2 — Supporting Information [file CTM2-11-e607-s002.docx]
